# Accurate Quantification and Characterization of Adeno-Associated Viral Vectors

**DOI:** 10.3389/fmicb.2019.01570

**Published:** 2019-07-17

**Authors:** David Dobnik, Polona Kogovšek, Tjaša Jakomin, Nejc Košir, Magda Tušek Žnidarič, Maja Leskovec, Stephen M. Kaminsky, Janet Mostrom, Hyunmi Lee, Maja Ravnikar

**Affiliations:** ^1^Department of Biotechnology and Systems Biology, National Institute of Biology, Ljubljana, Slovenia; ^2^BIA Separations, Ajdovščina, Slovenia; ^3^Belfer Gene Therapy Core Facility, Department of Genetic Medicine, Weill Medical College of Cornell University, New York, NY, United States

**Keywords:** absolute quantification, AAV, gene therapy, electron microscopy, digital PCR, real-time PCR

## Abstract

One of the main challenges in the gene therapy viral vector development is to establish an optimized process for its large scale production. This requires optimization for upstream and downstream processes as well as methods that enable the step-by step analytical characterization of the virus, the results of which inform the iterative refinement of production for yield, purity and potency. The biggest problem here is a plethora of viral vector formulations, many of which interfere with analytical techniques. We took adeno-associated virus (AAV) as an example and showed benefits of combined use of molecular methods and transmission electron microscopy (TEM) for viral vectors’ characterization and quantification. Results of the analyses showed that droplet digital PCR (ddPCR) performs better than quantitative real-time PCR (qPCR), in terms of robustness and assay variance, and this was especially relevant for partially purified (in-process) samples. Moreover, we demonstrate the importance of sample preparation prior to PCR analysis. We evaluated viral structure, presence of aggregates and impurities with TEM analysis and found that these impacted the differences in viral titers observed by qPCR and ddPCR and could be altered by sample preparation. These results serve as a guide for the establishment of the analytical methods required to provide measures of identity and purity for AAV viral vectors.

## Introduction

Adeno associated virus (AAV) is an important viral vector for gene therapy. It is useful due to its vast tropism, minimal immunogenicity, lack of association with any disease, and the capacity to achieve efficient and persistent gene transfer. AAV has limited packaging capability with about 5 kb of single stranded DNA. It would be the ideal gene transfer vector, if all cell types and tissues would be equally susceptible to AAV infection (Zinn and Vandenberghe, 2014). The first European approval of AAV-based gene therapy revealed the need for more efficient AAV vector manufacturing and downstream processing as the cost, among other reasons led to the product’s rapid commercial demise (Ai et al., 2017). Characterization and quantification are particular challenges in process development and production of viral vectors. Clinical dosing of recombinant AAV (rAAV) therapeutics are usually based on vector genome (vg) titer per mL, thus requiring availability of accurate quality control methods (D’Costa et al., 2016).

Multiple methods were and are still used for determination of vector genome titer. These include: dot-blot hybridization (Samulski et al., 1989) and Southern blotting (McCarty et al., 2001), which are not affected by secondary structure of construct terminal regions; UV spectrophotometry (Sommer et al., 2003) and PicoGreen based fluorimetry (Piedra et al., 2015), ELISA (Sondhi et al., 2005), and quantitative real-time PCR (qPCR) (D’Costa et al., 2016). A simple UV spectrophotometry assay can quantify both capsid protein and genomic DNA, but can be affected by cellular proteins and DNA impurities. qPCR has become the most widely used and accepted method for quantification of AAV vectors as it is simple and robust under ideal conditions. Its limitation is DNA amplification efficiency, which can be significantly impaired by different factors. These include poor design of primer pairs, presence of inhibitors, or secondary structure in the template, as noted for self-complimentary AAV vectors (Fagone et al., 2012), and the requirement of a valid DNA standard curve (Fagone et al., 2012; Lock et al., 2014; D’Costa et al., 2016), which can be incorrectly calibrated. Further, for validation of methods only two well characterized reference standards are currently available for AAV serotypes 2 and 8 (Moullier and Snyder, 2008; Lock et al., 2010; Ayuso et al., 2014).

Droplet digital PCR (ddPCR) has recently emerged as a powerful technique for absolute quantification of AAV. This method can eliminate the need for a standard curve and is less sensitive to inhibitors originating from components in the formulation (Pacouret et al., 2017). The total capsid content may be as important as the vector genome titer and to date there is no harmonized method to assay total capsids or the ability to distinguish empty particles from genome containing particles. Separation of AAV particles and quantification of full/empty ratio can be achieved using techniques, such as enzyme linked immunosorbent assay (ELISA), electron microscopy, analytical ultracentrifugation (AUC), and high pressure liquid chromatography (HPLC), where each has its own advantages and limitations (D’Costa et al., 2016; Dorange and Le Bec, 2018).

The aim of our study was to combine two approaches for the characterization of AAV vectors and to gather a more general view of specific AAV vector. We compared qPCR and ddPCR using different assays and pre-treatments to identify advantages and pitfalls of viral genome titering. We also provided a practical example showing indispensability of transmission electron microscopy (TEM) for visualizing AAV capsid integrity and impurities, as a complementary method to molecular methods.

## Materials and Methods

### AAV Material

AAVrh.10 vectors used in the study were composed of the Rhesus serotype 10 capsid proteins and the vector genome containing the AAV2 5′ inverted terminal repeat (ITR), the AAV2 packaging signal (C), CMV enhancer, chicken β-actin promoter and splice donor and rabbit β-globin intron with splice acceptor, the cDNA for the transgene followed by the rabbit β -globin polyA signal, and the AAV2 3′ ITR. AAVrh.10 were produced in HEK293T cells by co-transfecting the AAVrh.10 packaging-Ad helper hybrid plasmid pPAKMArh.10 and the transgene expression plasmid using PEIpro transfection reagent (Polyplus transfection, France). The cells expressing AAVrh.10 were harvested on the day 3 post transfection.

Two different AAVrh.10 vectors were used for this study. For the majority of experiments the test subject was AAVrh.10hCLN2 (Sondhi et al., 2005), but for the downstream purification testing the vector was AAVrh.10mCherry (Table 1).

**TABLE 1:** A list of performed experiments with corresponding test subjects, assays, pre-treatments, methods with number of replicates and statistical analysis.

**Experiment**	**Test subject**	**Assay**	**Pre-treatment**	**Methods with number of replicates**		**Statistical analysis**
Evaluation of assay variability and transfer from qPCR to ddPCR	AAVrh.10hCLN2	CMV	No pre-treatment Proteinase K DNase DNase+proteinase K	qPCR	One dilution (high concentration) in four replicates One dilution (low concentration) in four replicates	Average, SD
				ddPCR	Three dilutions each in two replicates (total six replicates)	
DNase concentration evaluation	AAVrh.10hCLN2	CMV	DNase	ddPCR	Two treatment replicates, two dilutions each in duplicate (total eight replicates)	Average, SD, CV
Testing partially purified samples	AAVrh.10mCherry	CMV	DNase+proteinase K DNase	qPCR	Three dilutions each in two replicates (total six replicates)	Average, SD, CV
				ddPCR	Three dilutions each in two replicates (total six replicates)	
Evaluations of different assays and pre-treatments	AAVrh.10hCLN2	CMV Beta actin Beta globin polyA	DNase DNase+denaturation DNase+denaturation+ITR restriction	qPCR	Three dilutions in triplicate (DNase) (total nine replicates) Six dilutions in triplicate (DNase+denaturation) (total 18 replicates) Four dilutions in triplicate (DNase+denaturation+ITR restriction) (total 12 replicates)	Average, SD, CV, ANOVA, Tukey’s multiple comparison of means
				ddPCR	Three dilutions in triplicate (DNase) (total nine replicates) Four dilutions in duplicate (total eight replicates)	
Evaluation of capsid integrity, presence of aggregates and impurities	AAVrh.10hCLN2	/	DNase	TEM	Two grids for each DNase concentration	/

### Virus Purification

#### AAVrh.10hCLN2

AAVrh.10hCLN2 was purified from the crude viral lysate by iodixanol gradient and QHP anion exchange chromatography (GE Healthcare, Piscataway, NJ, United States) (Supplementary Figure 1 in Supplementary Data Sheet 2). The purified vectors were concentrated using 100 kDa MWCO Amicon Ultra-15 centrifugal filter units (Millipore, Billerica, MA, United States) and stored in phosphate-buffered saline (PBS), pH 7.4, at −80°C.

#### Partially Purified Samples of AAVrh.10mCherry

HEK293T cells transfected to produce AAVrh.10mCherry were resuspended in lysis buffer, 20 mM Tris, 2 mM MgCl2, pH 8.0. Material was subsequently submitted to five freeze-thaw cycles. Freezing was done in dry ice/70% isopropanol slush and thawed in a water bath at 37°C (15 min). Lysate was clarified using laboratory centrifuge Sigma, 3K15 (Sigma Laborzentrifugen, DE, United States) for 35 min at 9384 *g* at 4°C. Cell debris was pelleted and supernatant was collected. Clarified lysate was used for chromatographic downstream process development on CIMmultus^*TM*^ monolithic columns (BIA Separations, Slovenia). CIMmultus^*TM*^ OH monolith or CIMmultus^*TM*^ SO3 column were flushed with water and equilibrated to 1.5 M potassium phosphate buffer (KPB), pH 7.0. rAAV lysate was diluted using potassium phosphate buffer (2 M KPB, pH 7.0) to achieve target concentration 1.5 M KPB. Sample was loaded onto the column and eluted with a linear gradient of 50 mM KPB pH 7.

### Assay Design

Four different assays were used for the quantification of AAV vectors used in the study, to cover the whole genome and enable to assess encapsidated genome integrity. The CMV qPCR assay was designed as described previously by Belfer Gene Therapy Core Facility at Weill Cornell Medicine (BGTCF) (Table 2; De et al., 2018). Three other assays, targeting beta-actin, beta-globin and polyA, were designed at National Institute of Biology (NIB) (Table 2). These were designed using Primer Express 2.0 software (Thermo Fisher Scientific, United States). TaqMan probe and primer design with default parameters were selected in the software. Sequence covering region of interest was inserted and software proposed several assays, from which the top scoring one was selected. Primers and probes were analyzed *in silico* using the AutoDimer software (Vallone and Butler, 2004). Probes labeled with FAM/BkFQ and primers were synthesized by Integrated DNA Technologies (Belgium).

**TABLE 2:** Primers and probes designed to quantify AAVrh.10hCLN2 and AAVrh.10-mCherry vector.

**Target**	**Label**	**DNA sequence of the oligonucleotide (5′-sequence-3′)**	**Final concentration in the reaction (nmol/L)**	**References**
CMV enhancer	FP-CMV	GTCAATGGGTGGAGTATTTACGG	900	De et al.,2018
	RP-CMV	GCATTATGCCCAGTACATGACCT	900	
	P-CMV	FAM-CAAGTGTATCATATGCCAAGTACGCCCCC-BkFQ	300	
Beta actin	FP-beta actin	CCGCAGCCATTGCCTTT	900	This study
	RP-beta actin	CCGCACAGATTTGGGACAA	900	
	P-beta actin	FAM-ATGGTAATCGTGCGAGAGGGCGC-BkFQ	300	
Beta globin	FP-beta globin	TCAGGTGCAGGCTGCCTAT	900	This study
	RP-beta globin	TTTGTGAGCCAGGGCATTG	900	
	P-beta globin	FAM-AGAAGGTGGTGGCTGGTGTGG-BkFQ	300	
PolyA	FP-polyA	GATTTTTCCTCCTCTCCTGACTACTC	900	This study
	RP-polyA	GCTGCAGGTCGAGGGATCT	900	
	P-polyA	FAM-CAGTCATAGCTGTCCCTCTTCTCTT-BkFQ	300	

*FP, forward primer; RP, reverse primer; P, probe; FAM, fluorescence reporter; BkFQ, black hole fluorescence quencher.*

### Pre-treatments of the Viral Vectors Prior to PCR

In PCR the samples were used directly (no pre-treatment) or were pre-treated with one, or combination of treatments described below. The list of pre-treatments used in each of the experiments is presented in Table 1.

Proteinase K treatment was initially employed for assessment of the CMV assay performance in ddPCR format and for testing of in process quality control samples consisting of partially purified AAV. The proteinase K reaction was composed of Proteinase K (Qiagen, Germany), 0.5 M EDTA (Sigma-Aldrich, United States), 10% SDS (Sigma-Aldrich, United States) and nuclease free water (NFW). The mixture was incubated at 55°C for 60 min in a thermoshaker, with shaking at 650 rpm. Proteinase K was inactivated with incubation at 95°C for 15 min.

Whenever DNase digestion protocol described by Lock et al. (2014) was used, it was always prior any other treatment and using only four units per reaction. The effect of DNase digestion on quantification of the vector by ddPCR was evaluated by testing six different concentrations of the enzyme (0, 2, 4, 8, 10, and 20 units per reaction) in the reaction volume of 200 μL, with 2 × 10^8^ expected encapsidated vector genomes per reaction. The DNase reaction mix was composed of Ambion DNase I Buffer (Thermo Fisher Scientific, United States), nuclease free water (NFW), sample and different concentrations of Ambion DNase I (Thermo Fisher Scientific, United States). The mixture was gently mixed and incubated at 37°C for 30 min.

Release of genomes from the capsid and release of secondary structure was performed with denaturation step, where samples were incubated at 95°C for 15 min and slowly cooled down to room temperature.

The ITR regions were cut from the vector, to reduce the stability of secondary structure after capsid denaturation. Vector restriction digest was performed with 0.5 μL of *Sal*I restriction enzyme (New England Biolabs, United States) and 22 μL of restriction buffer (New England Biolabs, United States). Reaction mixture was incubated at 37°C for 60 min followed by enzyme inactivation at 65°C for 20 min.

### Quantitative Real-Time PCR (qPCR)

Negative (NTC, no template control) and positive (known amount of the target DNA) controls were used in each run. Reaction mixtures in a final volume of 10 μL consisted of 5.5 μL of TaqMan 2× Universal PCR Master Mix (Life Technologies, United Kingdom), 0.5 μL of primers and probe mix (final concentration of primers was 900 nM and probes 250 nM), 2 μL of NFW and 2 μL of DNA or AAV template. Reaction mixtures were distributed in individual wells of 384-well plate, sealed and centrifuged. After centrifugation plate was transferred to 7900 HT Real-Time PCR system (Applied Biosystems, United States). Cycling conditions were 2 min at 50°C and 10 min at 95°C followed by 40 cycles of two step thermal profile composed of 15 s at 95°C and 60 s at 60°C.

The standard curve that enabled quantification of samples for comparison of protocols (samples 1–5 in Table 3) was prepared using denatured AAVrh.10hCLN2 vector with pre-defined concentration. It was composed of 6 sample points in triplicates that were prepared as 10× serial dilutions of original material. The material for qPCR standard curve was also characterized with ddPCR to increase the accuracy of quantification. For quantification of samples 6–14 (Table 3) a sample of AAVrh.10mCherry, characterized by ddPCR was used.

**TABLE 3:** Comparison of vector genome titer determined by qPCR and ddPCR on samples purified by CIM chromatography.

**Partially purified**		**qPCR**			**ddPCR**		
**downstream sample (number and treatment)**	**Buffer composition of the sample**	**Average quantity (vg/mL)**	**CV%**	**Bias of DNase to DNase+proteinase K**	**Average quantity (vg/mL)**	**CV%**	**Bias of DNase to DNase+proteinase K**
1 – DNase	1.5 mM potassium phosphate, pH 8	3.8E+11	12	−42%	8.9E+10	3	−19%
1 – DNase+Proteinase K		6.6E+11	17		1.1E+11	4	
2 – DNase	1.5 mM potassium phosphate, pH 8	2.6E+11	6	−54%	5.5E+10	5	−35%
2 – DNase+Proteinase K		5.6E+11	11		8.5E+10	7	
3 – DNase	1.5 mM potassium phosphate, pH 8	1.7E+11	20	−62%	4.8E+10	2	−27%
3 – DNase+Proteinase K		4.5E+11	6		6.6E+10	6^∗∗^	
4 – DNase	50 mM acetate, 100 mM NaCl, 0.5% Sucrose, 0.2% Poloxamer, pH 3.7	3.5E+10	10	−38%	6.7E+09	2^∗∗^	−17%
4 – DNase+Proteinase K		5.6E+10	7		8.1E+09	6	
5 – DNase	50 mM acetate, 100 mM NaCl, 0.5% Sucrose, 0.2% Poloxamer, pH 3.7	6.9E+10	10	−37%	1.5E+10	3	0%
5 – DNase+Proteinase K		1.1E+11	8		1.5E+10	3	
6 – DNase	20 mM Tris, 2 mM MgCl2, pH 8.0	4.4E+10	15	na	3.4E+10	7	na
7 – DNase	1.3 M potassium phosphate pH 7.0	3.0E+08^*^	42	na	4.1E+08	8	na
8 – DNase	0.7 M potassium phosphate pH 7.0	4.9E+09	15	na	4.2E+09	5	na
9 – DNase	1.5 M potassium phosphate pH 7.0	6.3E+09	23	na	5.8E+09	8	na
10 – DNase	1.5 M potassium phosphate pH 7.0	3.7E+08^*^	30	na	2.9E+08	12	na
11 – DNase	0.8 M potassium phosphate pH 7.0	1.6E+09	9	na	1.4E+09	8	na
12 – DNase	20 mM Tris, 2 mM MgCl_2_, pH 8.0	4.9E+10	9	na	4.8E+10	5	na
13 –DNase	1.1 M potassium phosphate pH 7.0	1.5E+10	9	na	1.2E+10	8	na
14 – DNase	1.1 M potassium phosphate pH 7.0	1.1E+09^*^	27	na	1.1E+09	12	na

Average quantity of the vector genome (vg/mL) was determined from nine to six replicates of qPCR and ddPCR, respectively, unless stated otherwise. ^*^First dilution used in experiment was inhibited (six replicates used for CV% calculation), ^∗∗^results for only one dilution due to saturated droplets (two replicates used for CV% calculation), na, not applicable.

Quantitative real-time PCR was used side-by-side with ddPCR in all experiments except of DNase concentration evaluation. Assessment of CMV assay variability was performed on two dilutions of untreated AAVrh.10hCLN2, which were later on used also as a second and third dilution for standard curve in testing of partially purified samples (termed as high and low concentration later on). In all other experiments quantification was performed based on the standard curve, as described above. Numbers of analyzed dilutions and technical replicates for each of the experiments are detailed in Table 1.

All the data were analyzed by SDS software version 2.4. Further analyses were performed in Microsoft Excel.

### Droplet Digital PCR (ddPCR)

Absolute quantification of vector genomes was performed using ddPCR. All experiments where ddPCR was used are presented in Table 1, which also includes information on numbers of dilutions and technical replicates for each of the experiments. Two of the three dilutions used for testing the CMV assay transfer in ddPCR were the same as in qPCR (high and low concentration), with one additional 10-fold dilution, which was also a part of qPCR standard curve.

Negative (NTC, no template control) and positive (known amount of the target DNA) controls were used in each run. The reaction mixture for ddPCR was composed of 10 μL 2× ddPCR Supermix for probes (no dUTP) (Bio-Rad, United States), 1 μL of primers and probe mix (final concentration of primers was 900 nM and probes 250 nM), 4 μL of NFW and 5 μL of the AAV sample. Five microliters of NFW was added instead of a DNA template as non-template control (NTC). A 10% surplus of each reaction mixture, including sample, was prepared and pipetted into each well of the 96-well plate (Eppendorf, Germany). Each sample was tested in duplicate and in three consecutive 10-fold dilutions. Plates were sealed, briefly vortexed, centrifuged and transferred to QX200 Automated Droplet Generator (Bio-Rad, United States) where droplets were automatically generated. 96-well plate (Eppendorf, Germany) with generated droplets was transferred to C1000 Touch Thermal Cycler (Bio-Rad, United States). Cycling conditions were 10 min at 95°C followed by 40 cycles of two step thermal profile composed of 30 s at 95°C and 60 s at 60°C at a ramp rate of 2.5°C/s. After cycling, plate was transferred to QX200 Droplet Reader (Bio-Rad, United States). Data analysis was performed using QuantaSoft software (version 1.7.4.0917; Bio-Rad, United States). Threshold separating negative and positive droplets was set manually just above the cluster of negative droplets. Data from wells with number of droplets bellow 8000 were rejected from analysis. Raw data were exported from QuantaSoft and further analyzed in Microsoft Excel. Copy numbers were calculated per μL, using a volume of 0.739 nL per droplet (Košir et al., 2017) to ensure the most accurate results.

### Secondary Structure Prediction

We conducted analysis of possible secondary structures of the vector construct sequence with Mfold, to explain observed differences in determined titers. Folding was analyzed at 60 and 99°C to identify different secondary structures of our construct. Other parameters were set to default.

### Transmission Electron Microscopy (TEM)

Transmission electron microscopy was used for observation of viral particles in starting material and also after DNase treatment in experiment to evaluate different DNase concentrations. 10–20 μL of viral (AAVrh.10hCLN2) suspension was applied on freshly glow-discharged copper grids (400 mesh, formvar-carbon coated) for 5 min, washed and stained with 1 droplet of 1% (w/v) water solution of uranyl acetate. Grids were prepared in duplicates. The grids were observed with transmission electron microscope Philips CM 100 (FEI, Netherlands), operating at 80 kV. At least 10 grid squares were examined thoroughly and representative micrographs (camera ORIUS SC 200, Gatan, Inc.) were taken at different magnifications.

### Statistical Analysis

The summary of used statistical analyses in performed PCR-based experiments is presented in Table 1. Average and standard deviation (SD) for each of the analyzed samples was calculated in Excel sheets from all of the sample replicates (dilutions and technical replicates). SD was also used for calculation of coefficient of variation. Statistical evaluation of differences between qPCR and ddPCR results of different assays and pre-treatments was performed by ANOVA followed by Tukey’s multiple comparison of means in R studio version 1.0.143. Differences in all coefficients of variation obtained after qPCR and ddPCR runs were evaluated by Student’s *t*-test in Excel.

## Results

In the presented study, we implemented a holistic approach and investigated critical points in AAV vector quantification and characterization. We evaluated transferability of assay from qPCR to ddPCR, compared both platforms and evaluated the effect of different sample pre-treatment protocols on quantification. We also showed the added value of implementing TEM analysis for viral vector characterization.

### Transfer of Established qPCR Protocols to ddPCR Is Possible Without Optimization

**FIGURE 1:**
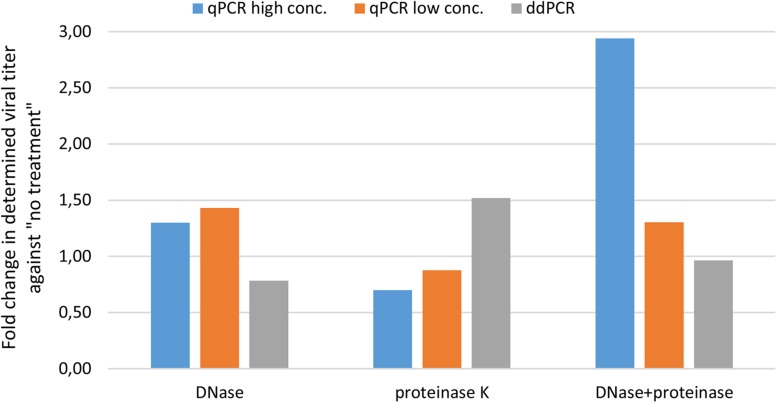
Evaluating variability of CMV assay results with different pre-treatments using qPCR and ddPCR. The difference in terms of fold change is presented for three pre-treatments against no treatment.

We first wanted to evaluate transferability of assay from qPCR to ddPCR. For this we took the qPCR assay targeting CMV enhancer and compare the extent of variability in obtained results on a AAVrh.10hCLN2 sample after transferring it to ddPCR (Table 1). In addition, we evaluated the effect of different options for pre-treatments of samples prior to qPCR and ddPCR, namely DNase digestion only, proteinase K treatment only, DNase digestion followed with proteinase K and no treatment (Table 1). qPCR results were evaluated individually for two dilutions of the initial sample (10-fold difference; high and low concentration). The variability of qPCR and ddPCR was assessed in terms of fold difference in genome quantity between minimum and maximum value of four replicates for individual pre-treatments, which ranged from 1.4× to 3.2× for qPCR and from 1.1× to 1.6× for ddPCR (Table 4). The fold difference between minimum and maximum value among different treatments was 7.7× for qPCR and 2.4× for ddPCR. Graphical presentation of qPCR and ddPCR fold change in genome titers of pre-treated samples against non-treated is presented in Figure 1.

**TABLE 4:** Fold difference between minimum and maximum genome titer of four qPCR and six ddPCR replicates for each pre-treatment.

	**qPCR high concentration**	**qPCR low concentration**	**ddPCR**
No treatment	2.2	1.6	1.6
DNase	1.8	2.3	1.2
Proteinase K	1.4	3.2	1.2
DNase+proteinase	2.3	1.7	1.1

*For qPCR two dilutions of a sample were tested (high and low concentration) in 4 replicates, whereas for ddPCR the results represent six replicates (three dilutions in duplicate).*

### DNase Digestion Is Recommended as Pre-treatment

**FIGURE 2:**
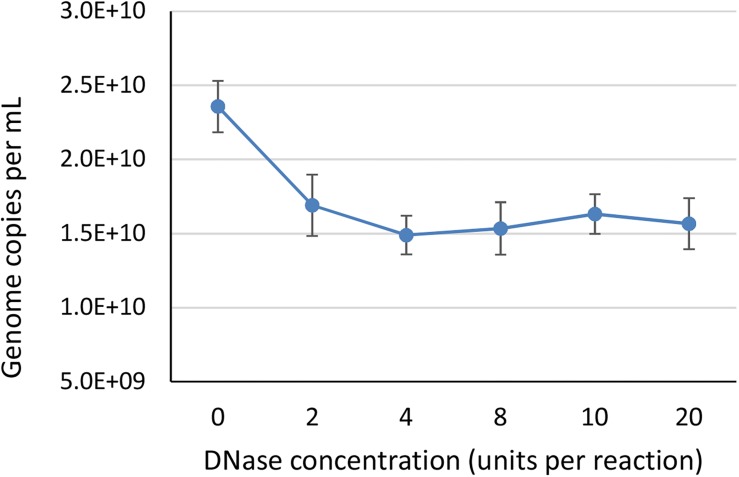
The effect of different DNase concentrations in units per 200 μL reaction, on the determined vector genome titer using ddPCR. Each DNase concentration was tested in two independent replicates, with two dilutions each in two technical replicates, resulting in total eight data points per DNase concentration. Error bars represent ±1 SD.

To identify the minimum required level of DNase enzyme for removal of non-encapsidated DNA, we tested six different concentrations of DNase (0, 2, 4, 8, 10, and 20 units per reaction) prior to ddPCR on a AAVrh.10hCLN2 sample using CMV assay (Table 1). We did not expect big differences between most of the treatments, thus we have conducted the study only with ddPCR due to its higher accuracy. We used a wide range of concentrations to observe any degradation of genomes that might be released from capsids as a result of proteinase activity, since bovine DNase I can include proteases as contaminants (McCarty, 1946). At the 2 unit level a significant drop in vector genome titer was observed (Figure 2), indicating the presence of free DNA. Further increase in DNase concentration did not have any significant effect on determined vector genome titer (Figure 2), indicating there was probably no non-specific proteinase activity of the DNAse used in the experiment.

### ddPCR Performs Better Than qPCR on AAVrh.10mCherry Samples at Interim Steps of the Downstream Purification

To test the effect of different buffer composition of samples on vector titering, we performed downstream purification of harvest lysate containing AAVrh.10mCherry with monolithic columns. We selected fourteen different fractions of samples coming from CIMmultus^*TM*^ chromatographic runs. Buffer composition in each of the tested samples is presented in Table 3. These samples were analyzed by qPCR and ddPCR using CMV assay, to evaluate, which method is more suitable for vector titering in downstream purification samples. Two different pre-treatments (only DNase digestion or DNase digestion with proteinase K) were used on a sub-set of five samples and nine samples were subjected only to DNase digestion. Quantification with qPCR showed 37–62% difference between determined average vector quantity of samples, when using DNase and proteinase K treatment in comparison to DNase only treatment (Table 3).

For the first set of samples (1–5 in Table 3), the determined qPCR titer based on the pre-defined concentration of standard curve material, was on average 64× higher than titer determined by ddPCR. When we used concentration of standard curve material as measured by ddPCR we observed a difference by a factor of 11.5× between pre-defined concentration with the measured one (1E+7 copies/mL considered in qPCR against 8.72E+5 copies/mL measured by ddPCR). By implementing this new concentration in the calculations (results presented in Table 3), the difference between qPCR and ddPCR values was reduced to the average factor of 5.5×. We observed inhibition of amplification in qPCR reaction for three samples (7, 10, 14) with high KPB concentration. For determining the titers of samples 6–14 (Table 3) a different standard curve material was used, but was also characterized by ddPCR to avoid too big difference. In this case the titers determined with qPCR were on average only 11% higher in comparison to ddPCR, which is within expected variation of these methods. Overall, the latter method was less affected by different treatments and showed smaller differences in determined titer in samples with different treatments (0–35% of bias). The coefficient of variation (CV%) between technical replicates was significantly lower in ddPCR, suggesting it is a more consistent method for analysis of intermediate up- and downstream samples.

### Pre-treatments and Assays Resulted in Different Vector Genome Titers

**FIGURE 3:**
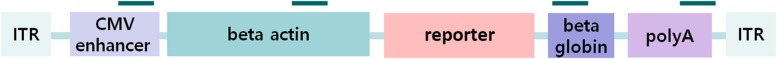
Schematic representation of annotated regions in AAVrh.10h vectors. The regions targeted by different assays are marked with short lines above the targeted gene.

More assays targeting a range of sequences within the AAVrh.10hCLN2 construct (Figure 3) provided a measure of vector DNA integrity and the influence of target position in the genome.

**FIGURE 4:**
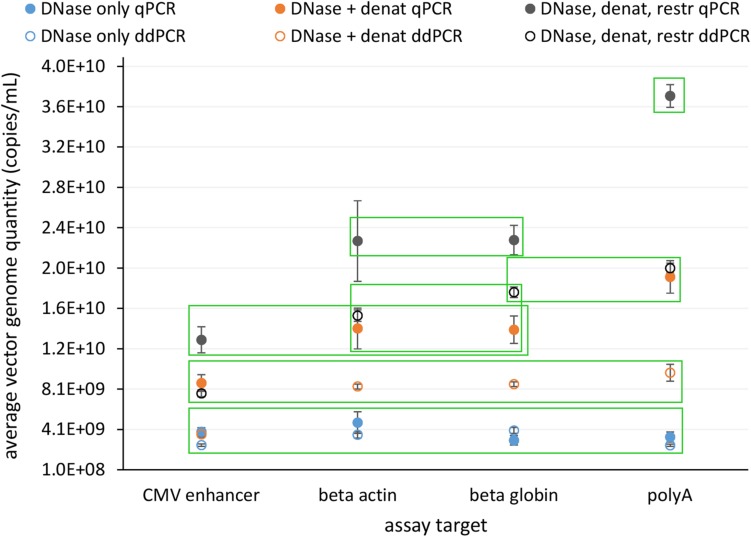
Average vector genome titer determined with four sequence targets (CMV enhancer, beta actin, beta globin, polyA) by qPCR and ddPCR after each of three different pre-treatments. Error bars represent ±1 SD. Filled dots are for qPCR and empty for ddPCR. Blue – DNase only, Orange – DNase and denaturation and Dark gray – DNase, denaturation and restriction. Each point represents at least eight replicates (see Table 1 for details on replicate number). Green squares enclose groups of results identified by statistical analysis (Tukey’s multiple comparison of means; *p* < 0.001).

In view of safety and efficacy of the gene therapy product, it is very important that the AAV capsids contain complete constructs. This can be examined using the assays targeting whole genome, where quantification results should be comparable for all. We tested four assays distributed over whole construct length and each targeting a different gene on the vector construct. We compared the response of all assays with qPCR and ddPCR on same starting material, which underwent different treatments prior to PCR: (1) only DNase digestion, (2) DNase digestion and denaturation of capsids, or (3) DNase digestion, denaturation of capsids and with restriction of ITR regions. Average vector genome quantities determined with four different assays for all of the treatments are summarized in Figure 4.

Statistical evaluation of differences between individual experiments identified seven statistically different groups with combinations of individual treatments and assays (Figure 4 and Supplementary Data Sheet 1).

The lowest vector genome titer was determined for samples treated only with DNase. This was also the only treatment with no significant differences in the determined vector genome titer over all assays and both PCR techniques (Figure 4). This same group also included DNase and denaturation treatment with ddPCR CMV assay. When DNase and denaturation treated samples were tested with assays targeting other regions of the construct (Beta actin, Beta globin, PolyA), higher titers were observed (Figure 4). Combination of all three treatments (DNase, denaturation, and restriction of ITR regions) further increased the resulting titer in each assay format (Figure 4).

We conducted analysis of possible secondary structures of the vector construct sequence with Mfold, to explain observations in differences in determined titers. The predicted secondary structure of the vector DNA was relatively complex at 60°C, with one bigger single stranded loop (Supplementary Figure 2 in Supplementary Data Sheet 2). This open loop was in the region of the beta globin and polyA assay, which when targeted by the PCR assay resulted in slightly higher titer, what could be a result of better accessibility. The predicted secondary structure of construct without ITR regions was different and less stable at 60°C (Supplementary Figure 3 in Supplementary Data Sheet 2). ITR regions were still predicted to be a double-stranded structure at 99°C (Supplementary Figure 4 in Supplementary Data Sheet 2).

### ddPCR Has Lower Coefficient of Variation

**FIGURE 5:**
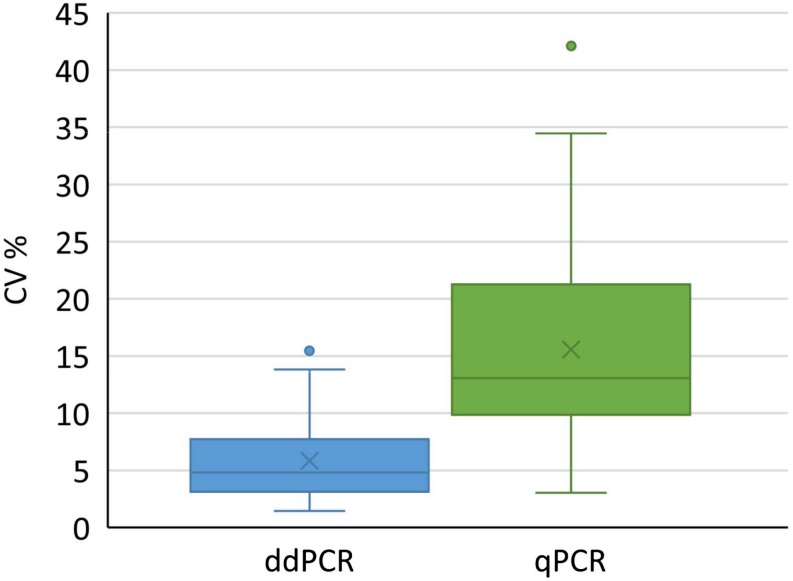
Distribution of coefficients of variation for results obtained with ddPCR and qPCR by testing 73 and 62 samples, respectively. Boxes represent the values between Q1 and Q3, whereas whiskers represent 1.5 interquartile range, horizontal lines represent medians, crosses represent average values. Individual points outside whiskers represent outliers. The CV% for each sample was calculated from at least six replicates. Results of both groups were significantly different (*t*-test, *p* < 0.001).

We used all conducted analyses and their results to evaluate the variability of determined coefficients of variation. We included different kinds of samples coming from downstream purification process, to cover the variety of possible samples in real-life testing. Altogether 73 and 63 samples were tested with ddPCR and qPCR, respectively. According to the analysis, a significantly lower coefficient of variation was observed with ddPCR compared to qPCR (Figure 5) across samples derived from distinct downstream purification steps. Therefore ddPCR is not only more precise, but is also a method of choice for samples that contain PCR inhibitors.

### TEM Analysis Identified Viral Aggregates and Damaged Capsids

AAVrh.10hCLN2 was observed with TEM to identify the impact of different pre-treatments on vector integrity and correlate this effect with quantification assays. We performed TEM analysis on the samples that were subjected to different DNase concentrations as a measure of degradation of viral capsids by high DNase concentration.

**FIGURE 6:**
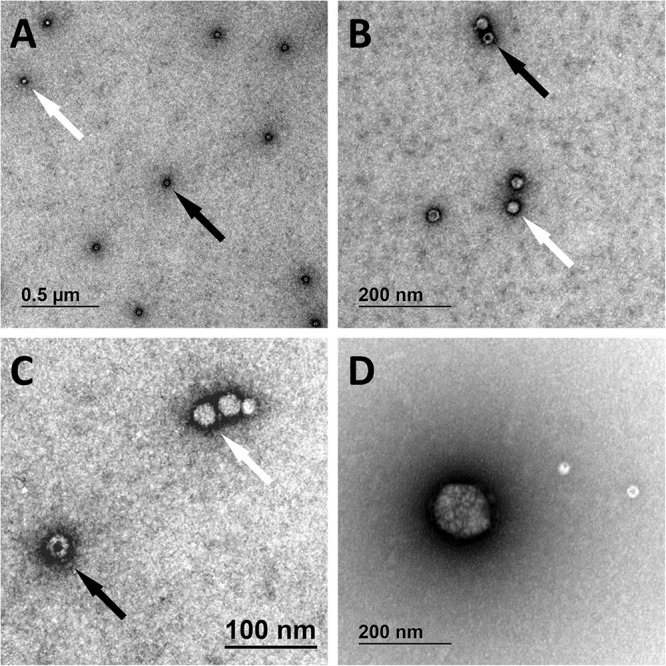
Transmission electron microscopy micrographs of AAVrh.10hCLN2. Full (bright spheres; white arrows) and empty (spheres with darker spot; black arrows) viral particles are shown under different magnifications **(A–C)**. Aggregate of viral particles is shown in panel **(D)**.

**FIGURE 7:**
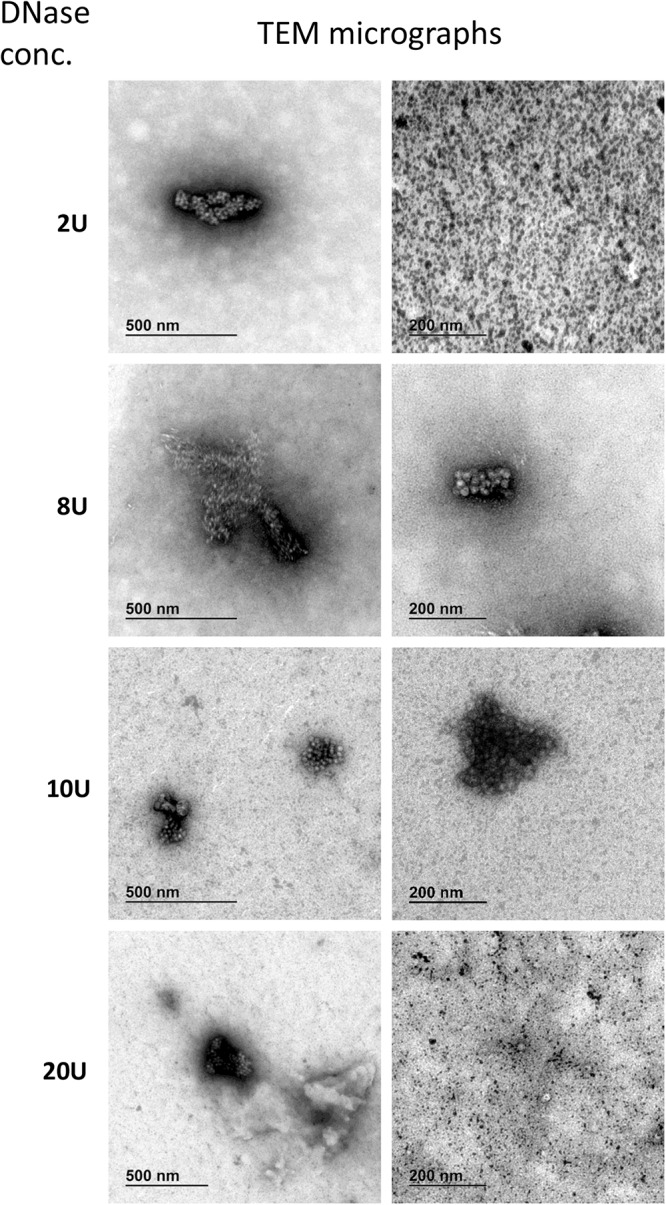
Transmission electron microscopy micrographs of AAVrh.10hCLN2 treated by different DNase concentrations (2, 8, 10, and 20 U). Two representative micrographs at different magnification are shown for each concentration of DNase. Aggregates of proteins (enzyme and/or virus particles) are visible on all micrographs, while no individual virus particles were observed.

We have identified full and empty viral particles (Figures 6A–C), smaller particles (Figure 6C) and some aggregates (Figure 6D), which probably also included damaged particles. In DNase treated samples most of viral particles were aggregated (Figure 7), which could be the consequence of non-optimal buffer for TEM analysis (DNase reaction buffer) or high protein content.

DNase treated samples were comparable in viral titer and aggregates content, independent of DNase concentration. No unique damaging effect to AAV capsids was observed due to DNase treatment. Additionally, we observed many protein aggregates or background in DNase treated samples (Figure 7). With increasing DNase concentration, there was an increase in the protein impurities and aggregates, probably a result of the aggregation of the DNase.

## Discussion

We employed different methods to get a more general overview of the specific AAV vector. Primary objective of our study was to demonstrate that different methods for quantification and characterization of AAV vectors should be used side-by-side, since the results of individual methods might be misleading. First, our focus was on comparison of qPCR and ddPCR, which are the common methods of choice for nucleic acid quantification. Our results exposed the need for specific sample pre-treatment and revealed the superiority of ddPCR in terms of robustness and quantification precision. We then combined those results with TEM analysis, which acted as a complementary method to molecular methods and explained some differences detected in PCR tests.

The transfer of CMV target qPCR protocol to ddPCR was easy. Treatment with only proteinase K resulted in higher titer compared to prior DNase digestion, due to the presence of free, non-encapsidated DNA. The use of DNase, already at two units per reaction, decreased the amount of detected vector genomes to a stable point where likely all free construct DNA was degraded. The stable titer throughout different concentrations of DNase indicated there was no proteinase activity of DNase I and therefore confirms the validity of including this step as described in Lock et al. (2014), but with lower DNase concentration than previously reported (Lock et al., 2010, 2014; Fagone et al., 2012).

To evaluate the utility of qPCR and ddPCR for titering in-process samples, given that qPCR was already shown to be comparable to ddPCR for purified AAV samples (Lock et al., 2010, 2014), AAVrh.10mCherry prepared from HEK293T cell derived cleared lysate, purified by CIMmultus^*TM*^ monolithic columns, was sampled at each of several steps in the downstream purification scheme. Buffers with high salt concentrations were used in the purification procedure. We observed inhibition of qPCR amplification in three samples, but no inhibition was observed with ddPCR. In those three samples the most probable cause of inhibition was high KPB concentration. Coefficient of variation was significantly lower for ddPCR in partially purified samples (Table 3) and also for all of the tested samples together (Figure 5). This indicates that ddPCR has better precision and thus presents a better choice for absolute quantification of in-process samples from the vector purification steps, in part due to its reduced sensitivity to formulation components. This is especially important for validation of assays for in-process control, where the validation should be performed for each matrix individually. ddPCR can be run without prior DNA extraction, which enables fast analysis time and can thus beneficial for fast determination of yield of downstream processes (Dorange and Le Bec, 2018). We have seen that there is actually no matrix interference with ddPCR by downstream (Table 3) or upstream samples (experience from contract research activities).

Use of assays that cover the whole genome best informs about the presence of an encapsidated whole genome. We compared the assays covering different regions of vector genome (Figure 3) using qPCR and ddPCR after different treatments, to check their possible interferences on vector genome titration. There were no differences in measured titer between the samples pre-treated with DNase. Previous reports suggested different amplification efficiencies for assays throughout AAV genome in qPCR (Fagone et al., 2012; Wang et al., 2013). Adding the denaturation step after DNase digestion already resulted in significant increase in titer determined by each of the several assays and PCR platforms (Figure 4). Furthermore, we prepared samples, in which after DNase digestion and denaturation the ITR regions were removed from the vector genome by restriction enzymes, to eliminate the possible influence of ITR regions. This increased the determined titer in some cases but not with all the assay methods (Figure 4). The predicted secondary structure for our vector was rather complex (Supplementary Figure 2 in Supplementary Data Sheet 2) but the open loop, containing target regions for beta globin and polyA assays is likely the reason for higher titer results using these two target sites. We could not explain all the differences between assays and pre-treatments by secondary structure. Thus we hypothesized that the viruses might be present in aggregates. Possible aggregates could cause biased results of ddPCR, since the partitioning of viral particles in droplets would not be random. Strong aggregation could also restrict access to genomes by qPCR. We examined the samples with TEM and confirmed the formation of aggregates, especially after the DNase reaction. It remains unclear whether the DNase damages particles, thus causing aggregation.

Adeno-associated virus sample/product, must be of sufficient quality/purity, if it is to be used in clinical trials or as final drug product. Visualizing approaches, such as TEM, are a necessity for assessment of sample integrity and purity in viral samples. Using TEM we were able to see individual viral particles and whether they were empty, full, damaged, aggregated, or of untypical size. This analysis helped us to explain our molecular results, which indicated something is happening in the particle level. TEM analysis was shown to be an essential part of viral vector characterization and we suggest its implementation in the regular quality control of viral vector production process. Nevertheless, only the information on full and/or empty capsids might not be sufficient, because what is actually inside capsids would be of bigger importance. AAVs can harbor relatively long DNA fragments and infection with intact particles containing unknown DNA sequence that became encapsidated during the production, can pose some health and regulatory concerns. Different high-throughput sequencing approaches could help to solve this question and shed more light on safety of AAV preparations.

Based on our previous work with ddPCR (Dreo et al., 2014; Pavšič et al., 2016; Dobnik et al., 2018), we expect that limits of detection and quantification would be comparable to qPCR. Inter-laboratory variability, as observed with qPCR (Lock et al., 2010; Ayuso et al., 2014), is likely smaller with ddPCR due to the lack of dependence on a standard curve and gold standard reference material. This would eliminate the need for common vector dosage unit mentioned by D’Costa et al. (2016), to which laboratories should calibrate their measurements.

Vector genomes can easily be detected and quantified by PCR, but they should be encapsidated to be infective. We did not address the infectivity of our vector in presented study. It was already shown that vector genome titer and infectious genome titer can be, and usually are, significantly different (Lock et al., 2010; Ayuso, 2016). It would be important to correlate this difference with either fragmented genome or damaged capsids in future studies.

The number of clinical trials using viral vectors is steadily increasing. Although there is yet no data to support this idea, we expect that the amount of viruses that might be released to the environment from the patients will increase following the future approvals of AAV gene therapies. This might pose some concerns, thus, environmental monitoring, such as is already in place for cytostatics, would probably need to be established. Detecting low concentration of viruses from environmental samples is challenging. Most definitely there is an issue of AAV stability in environment, which would need to be explored for AAV gene therapy preparations, before proceeding to environmental monitoring. We have shown that concentration and quantification of viruses from water (e.g., waste waters, rivers, sea) is possible (Rački et al., 2014a, b; Balasubramanian et al., 2016) and could also provide clear data on the amount of gene therapy viruses potentially released into the environment, once their stability is evaluated.

## Conclusion

In conclusion, we showed that characterization of viral vectors is much more informative with a combination of molecular approaches and microscopy, which provide orthogonal methods to characterize viral vectors. Our results serve as a guide for the establishment of the analytical testing scheme required to provide measures of identity and purity for AAV viral vectors. This is of special importance in the development of an optimized process for their large scale production. Any required optimization for upstream and downstream processes should be guided by selected combination of analytical methods that enable a step-by step analytical characterization of the virus, the results of which inform the iterative refinement of production for yield, purity and potency. To take a step further, we expect that emerging high-throughput sequencing technologies, such as Oxford Nanopore Technologies MinION sequencing, could offer additional information, which would nicely complement more traditional analytical approaches, and will most probably become a necessity in the future. ddPCR, TEM and high-throughput sequencing are thus definitely the combination of methods that should be used in AAV characterization process. Nevertheless, exact correlation between the outputs of different methods still needs to be established. Recently, the high-resolution capsid protein structure of AAV2 was reported (Tan et al., 2018). In addition to the gain of information by structural analysis we expect that future focus will also provide approaches for protein capsid integrity characterization, since this is a missing link between correlations of known analytical data with infectivity.

## Author Contributions

DD, PK, and MR designed the experiments. TJ, NK, MTŽ, and ML contributed to data acquisition. DD, PK, TJ, NK, MTŽ, and ML performed the data analysis. DD, PK, TJ, NK, MTŽ, and MR contributed to data interpretation. SK, JM, and HL prepared and provided both AAVrh.10 vectors and qPCR CMV enhancer protocol. DD wrote the first draft of the manuscript. PK, TJ, NK, and ML wrote sections of the manuscript. All authors read, critically revised the draft manuscript, and approved the submitted version.

## Conflict of Interest Statement

ML is employed in BIA Separations, a company that produces and sells CIM monolithic columns for concentration and purification of viruses. The remaining authors declare that the research was conducted in the absence of any commercial or financial relationships that could be construed as a potential conflict of interest.
