# Supplementary material for: Accurate Quantification and Characterization of Adeno-Associated Viral Vectors
Source: Front Microbiol. 2019 Jul 17;10:1570. doi: 10.3389/fmicb.2019.01570 (PMC6650692; doi:10.3389/fmicb.2019.01570)
Supplement: Supplementary file 1 [file Data_Sheet_1.pdf]

Supplementary Material

Accurate quantification and characterization of adeno-associated viral vectors

David Dobnik[*], Polona Kogovšek, Tjaša Jakomin, Nejc Košir, Magda Tušek Žnidarič, Maja Leskovec, Stephen M. Kaminsky, Janet Mostrom, Hyunmi Lee, Maja Ravnikar

*** Correspondence:**
David Dobnik: david.dobnik@nib.si

Data Sheet 1. A file with results of Tukey's multiple comparisons of means for all experimental groups with different pre-treatments and assays is available as separate file online.

Tukey multiple comparisons of means
95% family-wise confidence level

Compared results (PCR platform_pre-treatment_assay)

Group 1	Group 2	diff	lwr	upr
ddPCR_DNase_beta_actin	ddPCR_DNase_beta_globin	4,37E+08	-1,94E+09	2,81E+09
ddPCR_DNase_beta_actin	ddPCR_DNase_CMV_enhancer	-1,02E+09	-3,56E+09	1,53E+09
ddPCR_DNase_beta_actin	ddPCR_DNase_denat_beta_actin	4,80E+09	2,35E+09	7,25E+09
ddPCR_DNase_beta_actin	ddPCR_DNase_denat_beta_globin	5,02E+09	2,57E+09	7,47E+09
ddPCR_DNase_beta_actin	ddPCR_DNase_denat_CMV_enhancer	4,90E+07	-2,40E+09	2,50E+09
ddPCR_DNase_beta_actin	ddPCR_DNase_denat_polyA	6,16E+09	3,71E+09	8,61E+09
ddPCR_DNase_beta_actin	ddPCR_DNase_denat_restr_beta_actin	1,23E+10	9,87E+09	1,48E+10
ddPCR_DNase_beta_actin	ddPCR_DNase_denat_restr_beta_globin	1,41E+10	1,17E+10	1,66E+10
ddPCR_DNase_beta_actin	ddPCR_DNase_denat_restr_CMV_enhancer	4,12E+09	1,80E+09	6,43E+09
ddPCR_DNase_beta_actin	ddPCR_DNase_denat_restr_polyA	1,65E+10	1,41E+10	1,90E+10
ddPCR_DNase_beta_actin	ddPCR_DNase_polyA	-1,04E+09	-3,41E+09	1,34E+09
ddPCR_DNase_beta_actin	qPCR_DNase_beta_actin	1,21E+09	-1,16E+09	3,59E+09
ddPCR_DNase_beta_actin	qPCR_DNase_beta_globin	-3,49E+08	-2,72E+09	2,03E+09
ddPCR_DNase_beta_actin	qPCR_DNase_CMV_enhancer	-3,49E+08	-2,72E+09	2,03E+09
ddPCR_DNase_beta_actin	qPCR_DNase_denat_beta_actin	1,05E+10	8,51E+09	1,26E+10
ddPCR_DNase_beta_actin	qPCR_DNase_denat_beta_globin	1,04E+10	8,39E+09	1,25E+10
ddPCR_DNase_beta_actin	qPCR_DNase_denat_CMV_enhancer	5,14E+09	3,11E+09	7,18E+09
ddPCR_DNase_beta_actin	qPCR_DNase_denat_polyA	1,57E+10	1,36E+10	1,77E+10
ddPCR_DNase_beta_actin	qPCR_DNase_denat_restr_beta_actin	1,92E+10	1,70E+10	2,14E+10
ddPCR_DNase_beta_actin	qPCR_DNase_denat_restr_beta_globin	1,93E+10	1,71E+10	2,15E+10
ddPCR_DNase_beta_actin	qPCR_DNase_denat_restr_CMV_enhancer	9,42E+09	7,21E+09	1,16E+10
ddPCR_DNase_beta_actin	qPCR_DNase_denat_restr_polyA	3,36E+10	3,14E+10	3,58E+10
ddPCR_DNase_beta_actin	qPCR_DNase_polyA	-3,49E+08	-2,72E+09	2,03E+09
ddPCR_DNase_beta_globin	ddPCR_DNase_CMV_enhancer	-1,45E+09	-4,06E+09	1,15E+09
ddPCR_DNase_beta_globin	ddPCR_DNase_denat_beta_actin	4,36E+09	1,85E+09	6,87E+09
ddPCR_DNase_beta_globin	ddPCR_DNase_denat_beta_globin	4,59E+09	2,08E+09	7,10E+09
ddPCR_DNase_beta_globin	ddPCR_DNase_denat_CMV_enhancer	-3,88E+08	-2,90E+09	2,12E+09
ddPCR_DNase_beta_globin	ddPCR_DNase_denat_polyA	5,73E+09	3,21E+09	8,24E+09
ddPCR_DNase_beta_globin	ddPCR_DNase_denat_restr_beta_actin	1,19E+10	9,37E+09	1,44E+10
ddPCR_DNase_beta_globin	ddPCR_DNase_denat_restr_beta_globin	1,37E+10	1,12E+10	1,62E+10
ddPCR_DNase_beta_globin	ddPCR_DNase_denat_restr_CMV_enhancer	3,68E+09	1,30E+09	6,05E+09
ddPCR_DNase_beta_globin	ddPCR_DNase_denat_restr_polyA	1,61E+10	1,36E+10	1,86E+10
ddPCR_DNase_beta_globin	ddPCR_DNase_polyA	-1,47E+09	-3,91E+09	9,63E+08
ddPCR_DNase_beta_globin	qPCR_DNase_beta_actin	7,77E+08	-1,66E+09	3,21E+09
ddPCR_DNase_beta_globin	qPCR_DNase_beta_globin	-7,86E+08	-3,22E+09	1,65E+09
ddPCR_DNase_beta_globin	qPCR_DNase_CMV_enhancer	-7,86E+08	-3,22E+09	1,65E+09
ddPCR_DNase_beta_globin	qPCR_DNase_denat_beta_actin	1,01E+10	8,00E+09	1,22E+10
ddPCR_DNase_beta_globin	qPCR_DNase_denat_beta_globin	9,99E+09	7,88E+09	1,21E+10
ddPCR_DNase_beta_globin	qPCR_DNase_denat_CMV_enhancer	4,71E+09	2,60E+09	6,82E+09
ddPCR_DNase_beta_globin	qPCR_DNase_denat_polyA	1,52E+10	1,31E+10	1,73E+10
ddPCR_DNase_beta_globin	qPCR_DNase_denat_restr_beta_actin	1,88E+10	1,65E+10	2,11E+10
ddPCR_DNase_beta_globin	qPCR_DNase_denat_restr_beta_globin	1,89E+10	1,66E+10	2,12E+10
ddPCR_DNase_beta_globin	qPCR_DNase_denat_restr_CMV_enhancer	8,98E+09	6,71E+09	1,13E+10
ddPCR_DNase_beta_globin	qPCR_DNase_denat_restr_polyA	3,32E+10	3,09E+10	3,54E+10
ddPCR_DNase_beta_globin	qPCR_DNase_polyA	-7,86E+08	-3,22E+09	1,65E+09
ddPCR_DNase_CMV_enhancer	ddPCR_DNase_denat_beta_actin	5,81E+09	3,14E+09	8,49E+09
ddPCR_DNase_CMV_enhancer	ddPCR_DNase_denat_beta_globin	6,04E+09	3,37E+09	8,71E+09
ddPCR_DNase_CMV_enhancer	ddPCR_DNase_denat_CMV_enhancer	1,07E+09	-1,61E+09	3,74E+09
ddPCR_DNase_CMV_enhancer	ddPCR_DNase_denat_polyA	7,18E+09	4,50E+09	9,85E+09
ddPCR_DNase_CMV_enhancer	ddPCR_DNase_denat_restr_beta_actin	1,33E+10	1,07E+10	1,60E+10
ddPCR_DNase_CMV_enhancer	ddPCR_DNase_denat_restr_beta_globin	1,52E+10	1,25E+10	1,78E+10
ddPCR_DNase_CMV_enhancer	ddPCR_DNase_denat_restr_CMV_enhancer	5,13E+09	2,58E+09	7,68E+09
ddPCR_DNase_CMV_enhancer	ddPCR_DNase_denat_restr_polyA	1,76E+10	1,49E+10	2,02E+10
ddPCR_DNase_CMV_enhancer	ddPCR_DNase_polyA	-1,94E+07	-2,62E+09	2,58E+09
ddPCR_DNase_CMV_enhancer	qPCR_DNase_beta_actin	2,23E+09	-3,74E+08	4,83E+09
ddPCR_DNase_CMV_enhancer	qPCR_DNase_beta_globin	6,67E+08	-1,94E+09	3,27E+09
ddPCR_DNase_CMV_enhancer	qPCR_DNase_CMV_enhancer	6,67E+08	-1,94E+09	3,27E+09
ddPCR_DNase_CMV_enhancer	qPCR_DNase_denat_beta_actin	1,16E+10	9,26E+09	1,39E+10
ddPCR_DNase_CMV_enhancer	qPCR_DNase_denat_beta_globin	1,14E+10	9,14E+09	1,37E+10

ddPCR_DNase_CMV_enhancer	qPCR_DNase_denat_CMV_enhancer	6,16E+09	3,86E+09	8,46E+09
ddPCR_DNase_CMV_enhancer	qPCR_DNase_denat_polyA	1,67E+10	1,44E+10	1,90E+10
ddPCR_DNase_CMV_enhancer	qPCR_DNase_denat_restr_beta_actin	2,02E+10	1,78E+10	2,27E+10
ddPCR_DNase_CMV_enhancer	qPCR_DNase_denat_restr_beta_globin	2,03E+10	1,79E+10	2,28E+10
ddPCR_DNase_CMV_enhancer	qPCR_DNase_denat_restr_CMV_enhancer	1,04E+10	7,98E+09	1,29E+10
ddPCR_DNase_CMV_enhancer	qPCR_DNase_denat_restr_polyA	3,46E+10	3,22E+10	3,71E+10
ddPCR_DNase_CMV_enhancer	qPCR_DNase_polyA	6,67E+08	-1,94E+09	3,27E+09
ddPCR_DNase_denat_beta_actin	ddPCR_DNase_denat_beta_globin	2,27E+08	-2,36E+09	2,81E+09
ddPCR_DNase_denat_beta_actin	ddPCR_DNase_denat_CMV_enhancer	-4,75E+09	-7,33E+09	-2,16E+09
ddPCR_DNase_denat_beta_actin	ddPCR_DNase_denat_polyA	1,37E+09	-1,22E+09	3,95E+09
ddPCR_DNase_denat_beta_actin	ddPCR_DNase_denat_restr_beta_actin	7,52E+09	4,94E+09	1,01E+10
ddPCR_DNase_denat_beta_actin	ddPCR_DNase_denat_restr_beta_globin	9,34E+09	6,76E+09	1,19E+10
ddPCR_DNase_denat_beta_actin	ddPCR_DNase_denat_restr_CMV_enhancer	-6,82E+08	-3,13E+09	1,77E+09
ddPCR_DNase_denat_beta_actin	ddPCR_DNase_denat_restr_polyA	1,17E+10	9,16E+09	1,43E+10
ddPCR_DNase_denat_beta_actin	ddPCR_DNase_polyA	-5,83E+09	-8,34E+09	-3,32E+09
ddPCR_DNase_denat_beta_actin	qPCR_DNase_beta_actin	-3,58E+09	-6,09E+09	-1,07E+09
ddPCR_DNase_denat_beta_actin	qPCR_DNase_beta_globin	-5,15E+09	-7,66E+09	-2,64E+09
ddPCR_DNase_denat_beta_actin	qPCR_DNase_CMV_enhancer	-5,15E+09	-7,66E+09	-2,64E+09
ddPCR_DNase_denat_beta_actin	qPCR_DNase_denat_beta_actin	5,75E+09	3,55E+09	7,95E+09
ddPCR_DNase_denat_beta_actin	qPCR_DNase_denat_beta_globin	5,63E+09	3,44E+09	7,83E+09
ddPCR_DNase_denat_beta_actin	qPCR_DNase_denat_CMV_enhancer	3,47E+08	-1,85E+09	2,54E+09
ddPCR_DNase_denat_beta_actin	qPCR_DNase_denat_polyA	1,09E+10	8,66E+09	1,31E+10
ddPCR_DNase_denat_beta_actin	qPCR_DNase_denat_restr_beta_actin	1,44E+10	1,21E+10	1,68E+10
ddPCR_DNase_denat_beta_actin	qPCR_DNase_denat_restr_beta_globin	1,45E+10	1,22E+10	1,69E+10
ddPCR_DNase_denat_beta_actin	qPCR_DNase_denat_restr_CMV_enhancer	4,62E+09	2,27E+09	6,98E+09
ddPCR_DNase_denat_beta_actin	qPCR_DNase_denat_restr_polyA	2,88E+10	2,64E+10	3,12E+10
ddPCR_DNase_denat_beta_actin	qPCR_DNase_polyA	-5,15E+09	-7,66E+09	-2,64E+09
ddPCR_DNase_denat_beta_globin	ddPCR_DNase_denat_CMV_enhancer	-4,97E+09	-7,56E+09	-2,39E+09
ddPCR_DNase_denat_beta_globin	ddPCR_DNase_denat_polyA	1,14E+09	-1,44E+09	3,72E+09
ddPCR_DNase_denat_beta_globin	ddPCR_DNase_denat_restr_beta_actin	7,29E+09	4,71E+09	9,88E+09
ddPCR_DNase_denat_beta_globin	ddPCR_DNase_denat_restr_beta_globin	9,12E+09	6,53E+09	1,17E+10
ddPCR_DNase_denat_beta_globin	ddPCR_DNase_denat_restr_CMV_enhancer	-9,09E+08	-3,36E+09	1,54E+09
ddPCR_DNase_denat_beta_globin	ddPCR_DNase_denat_restr_polyA	1,15E+10	8,93E+09	1,41E+10
ddPCR_DNase_denat_beta_globin	ddPCR_DNase_polyA	-6,06E+09	-8,57E+09	-3,55E+09
ddPCR_DNase_denat_beta_globin	qPCR_DNase_beta_actin	-3,81E+09	-6,32E+09	-1,30E+09
ddPCR_DNase_denat_beta_globin	qPCR_DNase_beta_globin	-5,37E+09	-7,88E+09	-2,86E+09
ddPCR_DNase_denat_beta_globin	qPCR_DNase_CMV_enhancer	-5,37E+09	-7,88E+09	-2,86E+09
ddPCR_DNase_denat_beta_globin	qPCR_DNase_denat_beta_actin	5,52E+09	3,33E+09	7,72E+09
ddPCR_DNase_denat_beta_globin	qPCR_DNase_denat_beta_globin	5,40E+09	3,21E+09	7,60E+09
ddPCR_DNase_denat_beta_globin	qPCR_DNase_denat_CMV_enhancer	1,20E+08	-2,08E+09	2,32E+09
ddPCR_DNase_denat_beta_globin	qPCR_DNase_denat_polyA	1,06E+10	8,44E+09	1,28E+10
ddPCR_DNase_denat_beta_globin	qPCR_DNase_denat_restr_beta_actin	1,42E+10	1,18E+10	1,66E+10
ddPCR_DNase_denat_beta_globin	qPCR_DNase_denat_restr_beta_globin	1,43E+10	1,19E+10	1,66E+10
ddPCR_DNase_denat_beta_globin	qPCR_DNase_denat_restr_CMV_enhancer	4,40E+09	2,04E+09	6,76E+09
ddPCR_DNase_denat_beta_globin	qPCR_DNase_denat_restr_polyA	2,86E+10	2,62E+10	3,09E+10
ddPCR_DNase_denat_beta_globin	qPCR_DNase_polyA	-5,37E+09	-7,88E+09	-2,86E+09
ddPCR_DNase_denat_CMV_enhancer	ddPCR_DNase_denat_polyA	6,11E+09	3,53E+09	8,70E+09
ddPCR_DNase_denat_CMV_enhancer	ddPCR_DNase_denat_restr_beta_actin	1,23E+10	9,68E+09	1,49E+10
ddPCR_DNase_denat_CMV_enhancer	ddPCR_DNase_denat_restr_beta_globin	1,41E+10	1,15E+10	1,67E+10
ddPCR_DNase_denat_CMV_enhancer	ddPCR_DNase_denat_restr_CMV_enhancer	4,07E+09	1,62E+09	6,52E+09
ddPCR_DNase_denat_CMV_enhancer	ddPCR_DNase_denat_restr_polyA	1,65E+10	1,39E+10	1,91E+10
ddPCR_DNase_denat_CMV_enhancer	ddPCR_DNase_polyA	-1,08E+09	-3,60E+09	1,43E+09
ddPCR_DNase_denat_CMV_enhancer	qPCR_DNase_beta_actin	1,16E+09	-1,35E+09	3,68E+09
ddPCR_DNase_denat_CMV_enhancer	qPCR_DNase_beta_globin	-3,98E+08	-2,91E+09	2,11E+09
ddPCR_DNase_denat_CMV_enhancer	qPCR_DNase_CMV_enhancer	-3,98E+08	-2,91E+09	2,11E+09
ddPCR_DNase_denat_CMV_enhancer	qPCR_DNase_denat_beta_actin	1,05E+10	8,30E+09	1,27E+10
ddPCR_DNase_denat_CMV_enhancer	qPCR_DNase_denat_beta_globin	1,04E+10	8,18E+09	1,26E+10
ddPCR_DNase_denat_CMV_enhancer	qPCR_DNase_denat_CMV_enhancer	5,09E+09	2,90E+09	7,29E+09
ddPCR_DNase_denat_CMV_enhancer	qPCR_DNase_denat_polyA	1,56E+10	1,34E+10	1,78E+10
ddPCR_DNase_denat_CMV_enhancer	qPCR_DNase_denat_restr_beta_actin	1,92E+10	1,68E+10	2,15E+10
ddPCR_DNase_denat_CMV_enhancer	qPCR_DNase_denat_restr_beta_globin	1,93E+10	1,69E+10	2,16E+10
ddPCR_DNase_denat_CMV_enhancer	qPCR_DNase_denat_restr_CMV_enhancer	9,37E+09	7,01E+09	1,17E+10
ddPCR_DNase_denat_CMV_enhancer	qPCR_DNase_denat_restr_polyA	3,36E+10	3,12E+10	3,59E+10
ddPCR_DNase_denat_CMV_enhancer	qPCR_DNase_polyA	-3,98E+08	-2,91E+09	2,11E+09
ddPCR_DNase_denat_polyA	ddPCR_DNase_denat_restr_beta_actin	6,15E+09	3,57E+09	8,74E+09

ddPCR_DNase_denat_polyA	ddPCR_DNase_denat_restr_beta_globin	7,98E+09	5,39E+09	1,06E+10
ddPCR_DNase_denat_polyA	ddPCR_DNase_denat_restr_CMV_enhancer	-2,05E+09	-4,50E+09	4,04E+08
ddPCR_DNase_denat_polyA	ddPCR_DNase_denat_restr_polyA	1,04E+10	7,79E+09	1,30E+10
ddPCR_DNase_denat_polyA	ddPCR_DNase_polyA	-7,20E+09	-9,71E+09	-4,69E+09
ddPCR_DNase_denat_polyA	qPCR_DNase_beta_actin	-4,95E+09	-7,46E+09	-2,44E+09
ddPCR_DNase_denat_polyA	qPCR_DNase_beta_globin	-6,51E+09	-9,02E+09	-4,00E+09
ddPCR_DNase_denat_polyA	qPCR_DNase_CMV_enhancer	-6,51E+09	-9,02E+09	-4,00E+09
ddPCR_DNase_denat_polyA	qPCR_DNase_denat_beta_actin	4,38E+09	2,19E+09	6,58E+09
ddPCR_DNase_denat_polyA	qPCR_DNase_denat_beta_globin	4,27E+09	2,07E+09	6,46E+09
ddPCR_DNase_denat_polyA	qPCR_DNase_denat_CMV_enhancer	-1,02E+09	-3,21E+09	1,18E+09
ddPCR_DNase_denat_polyA	qPCR_DNase_denat_polyA	9,49E+09	7,30E+09	1,17E+10
ddPCR_DNase_denat_polyA	qPCR_DNase_denat_restr_beta_actin	1,31E+10	1,07E+10	1,54E+10
ddPCR_DNase_denat_polyA	qPCR_DNase_denat_restr_beta_globin	1,31E+10	1,08E+10	1,55E+10
ddPCR_DNase_denat_polyA	qPCR_DNase_denat_restr_CMV_enhancer	3,26E+09	9,00E+08	5,62E+09
ddPCR_DNase_denat_polyA	qPCR_DNase_denat_restr_polyA	2,74E+10	2,51E+10	2,98E+10
ddPCR_DNase_denat_polyA	qPCR_DNase_polyA	-6,51E+09	-9,02E+09	-4,00E+09
ddPCR_DNase_denat_restr_beta_actin	ddPCR_DNase_denat_restr_beta_globin	1,82E+09	-7,60E+08	4,41E+09
ddPCR_DNase_denat_restr_beta_actin	ddPCR_DNase_denat_restr_CMV_enhancer	-8,20E+09	-1,07E+10	-5,75E+09
ddPCR_DNase_denat_restr_beta_actin	ddPCR_DNase_denat_restr_polyA	4,22E+09	1,64E+09	6,80E+09
ddPCR_DNase_denat_restr_beta_actin	ddPCR_DNase_polyA	-1,34E+10	-1,59E+10	-1,08E+10
ddPCR_DNase_denat_restr_beta_actin	qPCR_DNase_beta_actin	-1,11E+10	-1,36E+10	-8,59E+09
ddPCR_DNase_denat_restr_beta_actin	qPCR_DNase_beta_globin	-1,27E+10	-1,52E+10	-1,02E+10
ddPCR_DNase_denat_restr_beta_actin	qPCR_DNase_CMV_enhancer	-1,27E+10	-1,52E+10	-1,02E+10
ddPCR_DNase_denat_restr_beta_actin	qPCR_DNase_denat_beta_actin	-1,77E+09	-3,96E+09	4,26E+08
ddPCR_DNase_denat_restr_beta_actin	qPCR_DNase_denat_beta_globin	-1,89E+09	-4,08E+09	3,07E+08
ddPCR_DNase_denat_restr_beta_actin	qPCR_DNase_denat_CMV_enhancer	-7,17E+09	-9,37E+09	-4,98E+09
ddPCR_DNase_denat_restr_beta_actin	qPCR_DNase_denat_polyA	3,34E+09	1,15E+09	5,54E+09
ddPCR_DNase_denat_restr_beta_actin	qPCR_DNase_denat_restr_beta_actin	6,90E+09	4,54E+09	9,26E+09
ddPCR_DNase_denat_restr_beta_actin	qPCR_DNase_denat_restr_beta_globin	7,00E+09	4,64E+09	9,35E+09
ddPCR_DNase_denat_restr_beta_actin	qPCR_DNase_denat_restr_CMV_enhancer	-2,89E+09	-5,25E+09	-5,36E+08
ddPCR_DNase_denat_restr_beta_actin	qPCR_DNase_denat_restr_polyA	2,13E+10	1,89E+10	2,36E+10
ddPCR_DNase_denat_restr_beta_actin	qPCR_DNase_polyA	-1,27E+10	-1,52E+10	-1,02E+10
ddPCR_DNase_denat_restr_beta_globin	ddPCR_DNase_denat_restr_CMV_enhancer	-1,00E+10	-1,25E+10	-7,57E+09
ddPCR_DNase_denat_restr_beta_globin	ddPCR_DNase_denat_restr_polyA	2,40E+09	-1,87E+08	4,98E+09
ddPCR_DNase_denat_restr_beta_globin	ddPCR_DNase_polyA	-1,52E+10	-1,77E+10	-1,27E+10
ddPCR_DNase_denat_restr_beta_globin	qPCR_DNase_beta_actin	-1,29E+10	-1,54E+10	-1,04E+10
ddPCR_DNase_denat_restr_beta_globin	qPCR_DNase_beta_globin	-1,45E+10	-1,70E+10	-1,20E+10
ddPCR_DNase_denat_restr_beta_globin	qPCR_DNase_CMV_enhancer	-1,45E+10	-1,70E+10	-1,20E+10
ddPCR_DNase_denat_restr_beta_globin	qPCR_DNase_denat_beta_actin	-3,59E+09	-5,79E+09	-1,40E+09
ddPCR_DNase_denat_restr_beta_globin	qPCR_DNase_denat_beta_globin	-3,71E+09	-5,91E+09	-1,52E+09
ddPCR_DNase_denat_restr_beta_globin	qPCR_DNase_denat_CMV_enhancer	-9,00E+09	-1,12E+10	-6,80E+09
ddPCR_DNase_denat_restr_beta_globin	qPCR_DNase_denat_polyA	1,52E+09	-6,78E+08	3,71E+09
ddPCR_DNase_denat_restr_beta_globin	qPCR_DNase_denat_restr_beta_actin	5,08E+09	2,72E+09	7,44E+09
ddPCR_DNase_denat_restr_beta_globin	qPCR_DNase_denat_restr_beta_globin	5,17E+09	2,81E+09	7,53E+09
ddPCR_DNase_denat_restr_beta_globin	qPCR_DNase_denat_restr_CMV_enhancer	-4,72E+09	-7,08E+09	-2,36E+09
ddPCR_DNase_denat_restr_beta_globin	qPCR_DNase_denat_restr_polyA	1,95E+10	1,71E+10	2,18E+10
ddPCR_DNase_denat_restr_beta_globin	qPCR_DNase_polyA	-1,45E+10	-1,70E+10	-1,20E+10
ddPCR_DNase_denat_restr_CMV_enhancer	ddPCR_DNase_denat_restr_polyA	1,24E+10	9,97E+09	1,49E+10
ddPCR_DNase_denat_restr_CMV_enhancer	ddPCR_DNase_polyA	-5,15E+09	-7,52E+09	-2,78E+09
ddPCR_DNase_denat_restr_CMV_enhancer	qPCR_DNase_beta_actin	-2,90E+09	-5,28E+09	-5,27E+08
ddPCR_DNase_denat_restr_CMV_enhancer	qPCR_DNase_beta_globin	-4,46E+09	-6,84E+09	-2,09E+09
ddPCR_DNase_denat_restr_CMV_enhancer	qPCR_DNase_CMV_enhancer	-4,46E+09	-6,84E+09	-2,09E+09
ddPCR_DNase_denat_restr_CMV_enhancer	qPCR_DNase_denat_beta_actin	6,43E+09	4,39E+09	8,47E+09
ddPCR_DNase_denat_restr_CMV_enhancer	qPCR_DNase_denat_beta_globin	6,31E+09	4,27E+09	8,35E+09
ddPCR_DNase_denat_restr_CMV_enhancer	qPCR_DNase_denat_CMV_enhancer	1,03E+09	-1,01E+09	3,07E+09
ddPCR_DNase_denat_restr_CMV_enhancer	qPCR_DNase_denat_polyA	1,15E+10	9,50E+09	1,36E+10
ddPCR_DNase_denat_restr_CMV_enhancer	qPCR_DNase_denat_restr_beta_actin	1,51E+10	1,29E+10	1,73E+10
ddPCR_DNase_denat_restr_CMV_enhancer	qPCR_DNase_denat_restr_beta_globin	1,52E+10	1,30E+10	1,74E+10
ddPCR_DNase_denat_restr_CMV_enhancer	qPCR_DNase_denat_restr_CMV_enhancer	5,31E+09	3,09E+09	7,52E+09
ddPCR_DNase_denat_restr_CMV_enhancer	qPCR_DNase_denat_restr_polyA	2,95E+10	2,73E+10	3,17E+10
ddPCR_DNase_denat_restr_CMV_enhancer	qPCR_DNase_polyA	-4,46E+09	-6,84E+09	-2,09E+09
ddPCR_DNase_denat_restr_polyA	ddPCR_DNase_polyA	-1,76E+10	-2,01E+10	-1,51E+10
ddPCR_DNase_denat_restr_polyA	qPCR_DNase_beta_actin	-1,53E+10	-1,78E+10	-1,28E+10
ddPCR_DNase_denat_restr_polyA	qPCR_DNase_beta_globin	-1,69E+10	-1,94E+10	-1,44E+10
ddPCR_DNase_denat_restr_polyA	qPCR_DNase_CMV_enhancer	-1,69E+10	-1,94E+10	-1,44E+10

ddPCR_DNase_denat_restr_polyA	qPCR_DNase_denat_beta_actin	-5,99E+09	-8,19E+09	-3,79E+09
ddPCR_DNase_denat_restr_polyA	qPCR_DNase_denat_beta_globin	-6,11E+09	-8,30E+09	-3,91E+09
ddPCR_DNase_denat_restr_polyA	qPCR_DNase_denat_CMV_enhancer	-1,14E+10	-1,36E+10	-9,20E+09
ddPCR_DNase_denat_restr_polyA	qPCR_DNase_denat_polyA	-8,79E+08	-3,07E+09	1,32E+09
ddPCR_DNase_denat_restr_polyA	qPCR_DNase_denat_restr_beta_actin	2,68E+09	3,21E+08	5,04E+09
ddPCR_DNase_denat_restr_polyA	qPCR_DNase_denat_restr_beta_globin	2,78E+09	4,17E+08	5,13E+09
ddPCR_DNase_denat_restr_polyA	qPCR_DNase_denat_restr_CMV_enhancer	-7,12E+09	-9,47E+09	-4,76E+09
ddPCR_DNase_denat_restr_polyA	qPCR_DNase_denat_restr_polyA	1,71E+10	1,47E+10	1,94E+10
ddPCR_DNase_denat_restr_polyA	qPCR_DNase_polyA	-1,69E+10	-1,94E+10	-1,44E+10
ddPCR_DNase_polyA	qPCR_DNase_beta_actin	2,25E+09	-1,87E+08	4,69E+09
ddPCR_DNase_polyA	qPCR_DNase_beta_globin	6,86E+08	-1,75E+09	3,12E+09
ddPCR_DNase_polyA	qPCR_DNase_CMV_enhancer	6,86E+08	-1,75E+09	3,12E+09
ddPCR_DNase_polyA	qPCR_DNase_denat_beta_actin	1,16E+10	9,47E+09	1,37E+10
ddPCR_DNase_polyA	qPCR_DNase_denat_beta_globin	1,15E+10	9,35E+09	1,36E+10
ddPCR_DNase_polyA	qPCR_DNase_denat_CMV_enhancer	6,18E+09	4,07E+09	8,29E+09
ddPCR_DNase_polyA	qPCR_DNase_denat_polyA	1,67E+10	1,46E+10	1,88E+10
ddPCR_DNase_polyA	qPCR_DNase_denat_restr_beta_actin	2,03E+10	1,80E+10	2,25E+10
ddPCR_DNase_polyA	qPCR_DNase_denat_restr_beta_globin	2,03E+10	1,81E+10	2,26E+10
ddPCR_DNase_polyA	qPCR_DNase_denat_restr_CMV_enhancer	1,05E+10	8,18E+09	1,27E+10
ddPCR_DNase_polyA	qPCR_DNase_denat_restr_polyA	3,46E+10	3,24E+10	3,69E+10
ddPCR_DNase_polyA	qPCR_DNase_polyA	6,86E+08	-1,75E+09	3,12E+09
qPCR_DNase_beta_actin	qPCR_DNase_beta_globin	-1,56E+09	-4,00E+09	8,73E+08
qPCR_DNase_beta_actin	qPCR_DNase_CMV_enhancer	-1,56E+09	-4,00E+09	8,73E+08
qPCR_DNase_beta_actin	qPCR_DNase_denat_beta_actin	9,33E+09	7,22E+09	1,14E+10
qPCR_DNase_beta_actin	qPCR_DNase_denat_beta_globin	9,21E+09	7,10E+09	1,13E+10
qPCR_DNase_beta_actin	qPCR_DNase_denat_CMV_enhancer	3,93E+09	1,82E+09	6,04E+09
qPCR_DNase_beta_actin	qPCR_DNase_denat_polyA	1,44E+10	1,23E+10	1,66E+10
qPCR_DNase_beta_actin	qPCR_DNase_denat_restr_beta_actin	1,80E+10	1,57E+10	2,03E+10
qPCR_DNase_beta_actin	qPCR_DNase_denat_restr_beta_globin	1,81E+10	1,58E+10	2,04E+10
qPCR_DNase_beta_actin	qPCR_DNase_denat_restr_CMV_enhancer	8,21E+09	5,93E+09	1,05E+10
qPCR_DNase_beta_actin	qPCR_DNase_denat_restr_polyA	3,24E+10	3,01E+10	3,47E+10
qPCR_DNase_beta_actin	qPCR_DNase_polyA	-1,56E+09	-4,00E+09	8,73E+08
qPCR_DNase_beta_globin	qPCR_DNase_CMV_enhancer	9,11E-05	-2,44E+09	2,44E+09
qPCR_DNase_beta_globin	qPCR_DNase_denat_beta_actin	1,09E+10	8,79E+09	1,30E+10
qPCR_DNase_beta_globin	qPCR_DNase_denat_beta_globin	1,08E+10	8,67E+09	1,29E+10
qPCR_DNase_beta_globin	qPCR_DNase_denat_CMV_enhancer	5,49E+09	3,38E+09	7,60E+09
qPCR_DNase_beta_globin	qPCR_DNase_denat_polyA	1,60E+10	1,39E+10	1,81E+10
qPCR_DNase_beta_globin	qPCR_DNase_denat_restr_beta_actin	1,96E+10	1,73E+10	2,18E+10
qPCR_DNase_beta_globin	qPCR_DNase_denat_restr_beta_globin	1,97E+10	1,74E+10	2,19E+10
qPCR_DNase_beta_globin	qPCR_DNase_denat_restr_CMV_enhancer	9,77E+09	7,49E+09	1,20E+10
qPCR_DNase_beta_globin	qPCR_DNase_denat_restr_polyA	3,40E+10	3,17E+10	3,62E+10
qPCR_DNase_beta_globin	qPCR_DNase_polyA	3,43E-05	-2,44E+09	2,44E+09
qPCR_DNase_CMV_enhancer	qPCR_DNase_denat_beta_actin	1,09E+10	8,79E+09	1,30E+10
qPCR_DNase_CMV_enhancer	qPCR_DNase_denat_beta_globin	1,08E+10	8,67E+09	1,29E+10
qPCR_DNase_CMV_enhancer	qPCR_DNase_denat_CMV_enhancer	5,49E+09	3,38E+09	7,60E+09
qPCR_DNase_CMV_enhancer	qPCR_DNase_denat_polyA	1,60E+10	1,39E+10	1,81E+10
qPCR_DNase_CMV_enhancer	qPCR_DNase_denat_restr_beta_actin	1,96E+10	1,73E+10	2,18E+10
qPCR_DNase_CMV_enhancer	qPCR_DNase_denat_restr_beta_globin	1,97E+10	1,74E+10	2,19E+10
qPCR_DNase_CMV_enhancer	qPCR_DNase_denat_restr_CMV_enhancer	9,77E+09	7,49E+09	1,20E+10
qPCR_DNase_CMV_enhancer	qPCR_DNase_denat_restr_polyA	3,40E+10	3,17E+10	3,62E+10
qPCR_DNase_CMV_enhancer	qPCR_DNase_polyA	-5,67E-05	-2,44E+09	2,44E+09
qPCR_DNase_denat_beta_actin	qPCR_DNase_denat_beta_globin	-1,19E+08	-1,84E+09	1,60E+09
qPCR_DNase_denat_beta_actin	qPCR_DNase_denat_CMV_enhancer	-5,40E+09	-7,13E+09	-3,68E+09
qPCR_DNase_denat_beta_actin	qPCR_DNase_denat_polyA	5,11E+09	3,39E+09	6,83E+09
qPCR_DNase_denat_beta_actin	qPCR_DNase_denat_restr_beta_actin	8,67E+09	6,74E+09	1,06E+10
qPCR_DNase_denat_beta_actin	qPCR_DNase_denat_restr_beta_globin	8,76E+09	6,84E+09	1,07E+10
qPCR_DNase_denat_beta_actin	qPCR_DNase_denat_restr_CMV_enhancer	-1,13E+09	-3,05E+09	8,00E+08
qPCR_DNase_denat_beta_actin	qPCR_DNase_denat_restr_polyA	2,31E+10	2,11E+10	2,50E+10
qPCR_DNase_denat_beta_actin	qPCR_DNase_polyA	-1,09E+10	-1,30E+10	-8,79E+09
qPCR_DNase_denat_beta_globin	qPCR_DNase_denat_CMV_enhancer	-5,28E+09	-7,01E+09	-3,56E+09
qPCR_DNase_denat_beta_globin	qPCR_DNase_denat_polyA	5,23E+09	3,51E+09	6,95E+09
qPCR_DNase_denat_beta_globin	qPCR_DNase_denat_restr_beta_actin	8,79E+09	6,86E+09	1,07E+10
qPCR_DNase_denat_beta_globin	qPCR_DNase_denat_restr_beta_globin	8,88E+09	6,96E+09	1,08E+10
qPCR_DNase_denat_beta_globin	qPCR_DNase_denat_restr_CMV_enhancer	-1,01E+09	-2,93E+09	9,19E+08
qPCR_DNase_denat_beta_globin	qPCR_DNase_denat_restr_polyA	2,32E+10	2,12E+10	2,51E+10

qPCR_DNase_denat_beta_globin	qPCR_DNase_polyA	-1,08E+10	-1,29E+10	-8,67E+09
qPCR_DNase_denat_CMV_enhancer	qPCR_DNase_denat_polyA	1,05E+10	8,79E+09	1,22E+10
qPCR_DNase_denat_CMV_enhancer	qPCR_DNase_denat_restr_beta_actin	1,41E+10	1,21E+10	1,60E+10
qPCR_DNase_denat_CMV_enhancer	qPCR_DNase_denat_restr_beta_globin	1,42E+10	1,22E+10	1,61E+10
qPCR_DNase_denat_CMV_enhancer	qPCR_DNase_denat_restr_CMV_enhancer	4,28E+09	2,35E+09	6,20E+09
qPCR_DNase_denat_CMV_enhancer	qPCR_DNase_denat_restr_polyA	2,85E+10	2,65E+10	3,04E+10
qPCR_DNase_denat_CMV_enhancer	qPCR_DNase_polyA	-5,49E+09	-7,60E+09	-3,38E+09
qPCR_DNase_denat_polyA	qPCR_DNase_denat_restr_beta_actin	3,56E+09	1,63E+09	5,48E+09
qPCR_DNase_denat_polyA	qPCR_DNase_denat_restr_beta_globin	3,65E+09	1,73E+09	5,58E+09
qPCR_DNase_denat_polyA	qPCR_DNase_denat_restr_CMV_enhancer	-6,24E+09	-8,16E+09	-4,31E+09
qPCR_DNase_denat_polyA	qPCR_DNase_denat_restr_polyA	1,79E+10	1,60E+10	1,99E+10
qPCR_DNase_denat_polyA	qPCR_DNase_polyA	-1,60E+10	-1,81E+10	-1,39E+10
qPCR_DNase_denat_restr_beta_actin	qPCR_DNase_denat_restr_beta_globin	9,53E+07	-2,01E+09	2,20E+09
qPCR_DNase_denat_restr_beta_actin	qPCR_DNase_denat_restr_CMV_enhancer	-9,80E+09	-1,19E+10	-7,69E+09
qPCR_DNase_denat_restr_beta_actin	qPCR_DNase_denat_restr_polyA	1,44E+10	1,23E+10	1,65E+10
qPCR_DNase_denat_restr_beta_actin	qPCR_DNase_polyA	-1,96E+10	-2,18E+10	-1,73E+10
qPCR_DNase_denat_restr_beta_globin	qPCR_DNase_denat_restr_CMV_enhancer	-9,89E+09	-1,20E+10	-7,78E+09
qPCR_DNase_denat_restr_beta_globin	qPCR_DNase_denat_restr_polyA	1,43E+10	1,22E+10	1,64E+10
qPCR_DNase_denat_restr_beta_globin	qPCR_DNase_polyA	-1,97E+10	-2,19E+10	-1,74E+10
qPCR_DNase_denat_restr_CMV_enhancer	qPCR_DNase_denat_restr_polyA	2,42E+10	2,21E+10	2,63E+10
qPCR_DNase_denat_restr_CMV_enhancer	qPCR_DNase_polyA	-9,77E+09	-1,20E+10	-7,49E+09
qPCR_DNase_denat_restr_polyA	qPCR_DNase_polyA	-3,40E+10	-3,62E+10	-3,17E+10

		adjusted p value
ddPCR_DNase_beta_actin	ddPCR_DNase_beta_globin	1,00000
ddPCR_DNase_beta_actin	ddPCR_DNase_CMV_enhancer	0,99879
ddPCR_DNase_beta_actin	ddPCR_DNase_denat_beta_actin	0,00000
ddPCR_DNase_beta_actin	ddPCR_DNase_denat_beta_globin	0,00000
ddPCR_DNase_beta_actin	ddPCR_DNase_denat_CMV_enhancer	1,00000
ddPCR_DNase_beta_actin	ddPCR_DNase_denat_polyA	0,00000
ddPCR_DNase_beta_actin	ddPCR_DNase_denat_restr_beta_actin	0,00000
ddPCR_DNase_beta_actin	ddPCR_DNase_denat_restr_beta_globin	0,00000
ddPCR_DNase_beta_actin	ddPCR_DNase_denat_restr_CMV_enhancer	0,00000
ddPCR_DNase_beta_actin	ddPCR_DNase_denat_restr_polyA	0,00000
ddPCR_DNase_beta_actin	ddPCR_DNase_polyA	0,99573
ddPCR_DNase_beta_actin	qPCR_DNase_beta_actin	0,96992
ddPCR_DNase_beta_actin	qPCR_DNase_beta_globin	1,00000
ddPCR_DNase_beta_actin	qPCR_DNase_CMV_enhancer	1,00000
ddPCR_DNase_beta_actin	qPCR_DNase_denat_beta_actin	0,00000
ddPCR_DNase_beta_actin	qPCR_DNase_denat_beta_globin	0,00000
ddPCR_DNase_beta_actin	qPCR_DNase_denat_CMV_enhancer	0,00000
ddPCR_DNase_beta_actin	qPCR_DNase_denat_polyA	0,00000
ddPCR_DNase_beta_actin	qPCR_DNase_denat_restr_beta_actin	0,00000
ddPCR_DNase_beta_actin	qPCR_DNase_denat_restr_beta_globin	0,00000
ddPCR_DNase_beta_actin	qPCR_DNase_denat_restr_CMV_enhancer	0,00000
ddPCR_DNase_beta_actin	qPCR_DNase_denat_restr_polyA	0,00000
ddPCR_DNase_beta_actin	qPCR_DNase_polyA	1,00000
ddPCR_DNase_beta_globin	ddPCR_DNase_CMV_enhancer	0,92667
ddPCR_DNase_beta_globin	ddPCR_DNase_denat_beta_actin	0,00000
ddPCR_DNase_beta_globin	ddPCR_DNase_denat_beta_globin	0,00000
ddPCR_DNase_beta_globin	ddPCR_DNase_denat_CMV_enhancer	1,00000
ddPCR_DNase_beta_globin	ddPCR_DNase_denat_polyA	0,00000
ddPCR_DNase_beta_globin	ddPCR_DNase_denat_restr_beta_actin	0,00000
ddPCR_DNase_beta_globin	ddPCR_DNase_denat_restr_beta_globin	0,00000
ddPCR_DNase_beta_globin	ddPCR_DNase_denat_restr_CMV_enhancer	0,00001
ddPCR_DNase_beta_globin	ddPCR_DNase_denat_restr_polyA	0,00000
ddPCR_DNase_beta_globin	ddPCR_DNase_polyA	0,85324
ddPCR_DNase_beta_globin	qPCR_DNase_beta_actin	0,99997
ddPCR_DNase_beta_globin	qPCR_DNase_beta_globin	0,99996
ddPCR_DNase_beta_globin	qPCR_DNase_CMV_enhancer	0,99996
ddPCR_DNase_beta_globin	qPCR_DNase_denat_beta_actin	0,00000
ddPCR_DNase_beta_globin	qPCR_DNase_denat_beta_globin	0,00000
ddPCR_DNase_beta_globin	qPCR_DNase_denat_CMV_enhancer	0,00000
ddPCR_DNase_beta_globin	qPCR_DNase_denat_polyA	0,00000
ddPCR_DNase_beta_globin	qPCR_DNase_denat_restr_beta_actin	0,00000

ddPCR_DNase_beta_globin	qPCR_DNase_denat_restr_beta_globin	0,00000
ddPCR_DNase_beta_globin	qPCR_DNase_denat_restr_CMV_enhancer	0,00000
ddPCR_DNase_beta_globin	qPCR_DNase_denat_restr_polyA	0,00000
ddPCR_DNase_beta_globin	qPCR_DNase_polyA	0,99996
ddPCR_DNase_CMV_enhancer	ddPCR_DNase_denat_beta_actin	0,00000
ddPCR_DNase_CMV_enhancer	ddPCR_DNase_denat_beta_globin	0,00000
ddPCR_DNase_CMV_enhancer	ddPCR_DNase_denat_CMV_enhancer	0,99882
ddPCR_DNase_CMV_enhancer	ddPCR_DNase_denat_polyA	0,00000
ddPCR_DNase_CMV_enhancer	ddPCR_DNase_denat_restr_beta_actin	0,00000
ddPCR_DNase_CMV_enhancer	ddPCR_DNase_denat_restr_beta_globin	0,00000
ddPCR_DNase_CMV_enhancer	ddPCR_DNase_denat_restr_CMV_enhancer	0,00000
ddPCR_DNase_CMV_enhancer	ddPCR_DNase_denat_restr_polyA	0,00000
ddPCR_DNase_CMV_enhancer	ddPCR_DNase_polyA	1,00000
ddPCR_DNase_CMV_enhancer	qPCR_DNase_beta_actin	0,21643
ddPCR_DNase_CMV_enhancer	qPCR_DNase_beta_globin	1,00000
ddPCR_DNase_CMV_enhancer	qPCR_DNase_CMV_enhancer	1,00000
ddPCR_DNase_CMV_enhancer	qPCR_DNase_denat_beta_actin	0,00000
ddPCR_DNase_CMV_enhancer	qPCR_DNase_denat_beta_globin	0,00000
ddPCR_DNase_CMV_enhancer	qPCR_DNase_denat_CMV_enhancer	0,00000
ddPCR_DNase_CMV_enhancer	qPCR_DNase_denat_polyA	0,00000
ddPCR_DNase_CMV_enhancer	qPCR_DNase_denat_restr_beta_actin	0,00000
ddPCR_DNase_CMV_enhancer	qPCR_DNase_denat_restr_beta_globin	0,00000
ddPCR_DNase_CMV_enhancer	qPCR_DNase_denat_restr_CMV_enhancer	0,00000
ddPCR_DNase_CMV_enhancer	qPCR_DNase_denat_restr_polyA	0,00000
ddPCR_DNase_CMV_enhancer	qPCR_DNase_polyA	1,00000
ddPCR_DNase_denat_beta_actin	ddPCR_DNase_denat_beta_globin	1,00000
ddPCR_DNase_denat_beta_actin	ddPCR_DNase_denat_CMV_enhancer	0,00000
ddPCR_DNase_denat_beta_actin	ddPCR_DNase_denat_polyA	0,95702
ddPCR_DNase_denat_beta_actin	ddPCR_DNase_denat_restr_beta_actin	0,00000
ddPCR_DNase_denat_beta_actin	ddPCR_DNase_denat_restr_beta_globin	0,00000
ddPCR_DNase_denat_beta_actin	ddPCR_DNase_denat_restr_CMV_enhancer	1,00000
ddPCR_DNase_denat_beta_actin	ddPCR_DNase_denat_restr_polyA	0,00000
ddPCR_DNase_denat_beta_actin	ddPCR_DNase_polyA	0,00000
ddPCR_DNase_denat_beta_actin	qPCR_DNase_beta_actin	0,00009
ddPCR_DNase_denat_beta_actin	qPCR_DNase_beta_globin	0,00000
ddPCR_DNase_denat_beta_actin	qPCR_DNase_CMV_enhancer	0,00000
ddPCR_DNase_denat_beta_actin	qPCR_DNase_denat_beta_actin	0,00000
ddPCR_DNase_denat_beta_actin	qPCR_DNase_denat_beta_globin	0,00000
ddPCR_DNase_denat_beta_actin	qPCR_DNase_denat_CMV_enhancer	1,00000
ddPCR_DNase_denat_beta_actin	qPCR_DNase_denat_polyA	0,00000
ddPCR_DNase_denat_beta_actin	qPCR_DNase_denat_restr_beta_actin	0,00000
ddPCR_DNase_denat_beta_actin	qPCR_DNase_denat_restr_beta_globin	0,00000
ddPCR_DNase_denat_beta_actin	qPCR_DNase_denat_restr_CMV_enhancer	0,00000
ddPCR_DNase_denat_beta_actin	qPCR_DNase_denat_restr_polyA	0,00000
ddPCR_DNase_denat_beta_actin	qPCR_DNase_polyA	0,00000
ddPCR_DNase_denat_beta_globin	ddPCR_DNase_denat_CMV_enhancer	0,00000
ddPCR_DNase_denat_beta_globin	ddPCR_DNase_denat_polyA	0,99507
ddPCR_DNase_denat_beta_globin	ddPCR_DNase_denat_restr_beta_actin	0,00000
ddPCR_DNase_denat_beta_globin	ddPCR_DNase_denat_restr_beta_globin	0,00000
ddPCR_DNase_denat_beta_globin	ddPCR_DNase_denat_restr_CMV_enhancer	0,99961
ddPCR_DNase_denat_beta_globin	ddPCR_DNase_denat_restr_polyA	0,00000
ddPCR_DNase_denat_beta_globin	ddPCR_DNase_polyA	0,00000
ddPCR_DNase_denat_beta_globin	qPCR_DNase_beta_actin	0,00002
ddPCR_DNase_denat_beta_globin	qPCR_DNase_beta_globin	0,00000
ddPCR_DNase_denat_beta_globin	qPCR_DNase_CMV_enhancer	0,00000
ddPCR_DNase_denat_beta_globin	qPCR_DNase_denat_beta_actin	0,00000
ddPCR_DNase_denat_beta_globin	qPCR_DNase_denat_beta_globin	0,00000
ddPCR_DNase_denat_beta_globin	qPCR_DNase_denat_CMV_enhancer	1,00000
ddPCR_DNase_denat_beta_globin	qPCR_DNase_denat_polyA	0,00000
ddPCR_DNase_denat_beta_globin	qPCR_DNase_denat_restr_beta_actin	0,00000
ddPCR_DNase_denat_beta_globin	qPCR_DNase_denat_restr_beta_globin	0,00000
ddPCR_DNase_denat_beta_globin	qPCR_DNase_denat_restr_CMV_enhancer	0,00000
ddPCR_DNase_denat_beta_globin	qPCR_DNase_denat_restr_polyA	0,00000
ddPCR_DNase_denat_beta_globin	qPCR_DNase_polyA	0,00000
ddPCR_DNase_denat_CMV_enhancer	ddPCR_DNase_denat_polyA	0,00000

ddPCR_DNase_denat_CMV_enhancer	ddPCR_DNase_denat_restr_beta_actin	0,00000
ddPCR_DNase_denat_CMV_enhancer	ddPCR_DNase_denat_restr_beta_globin	0,00000
ddPCR_DNase_denat_CMV_enhancer	ddPCR_DNase_denat_restr_CMV_enhancer	0,00000
ddPCR_DNase_denat_CMV_enhancer	ddPCR_DNase_denat_restr_polyA	0,00000
ddPCR_DNase_denat_CMV_enhancer	ddPCR_DNase_polyA	0,99626
ddPCR_DNase_denat_CMV_enhancer	qPCR_DNase_beta_actin	0,99043
ddPCR_DNase_denat_CMV_enhancer	qPCR_DNase_beta_globin	1,00000
ddPCR_DNase_denat_CMV_enhancer	qPCR_DNase_CMV_enhancer	1,00000
ddPCR_DNase_denat_CMV_enhancer	qPCR_DNase_denat_beta_actin	0,00000
ddPCR_DNase_denat_CMV_enhancer	qPCR_DNase_denat_beta_globin	0,00000
ddPCR_DNase_denat_CMV_enhancer	qPCR_DNase_denat_CMV_enhancer	0,00000
ddPCR_DNase_denat_CMV_enhancer	qPCR_DNase_denat_polyA	0,00000
ddPCR_DNase_denat_CMV_enhancer	qPCR_DNase_denat_restr_beta_actin	0,00000
ddPCR_DNase_denat_CMV_enhancer	qPCR_DNase_denat_restr_beta_globin	0,00000
ddPCR_DNase_denat_CMV_enhancer	qPCR_DNase_denat_restr_CMV_enhancer	0,00000
ddPCR_DNase_denat_CMV_enhancer	qPCR_DNase_denat_restr_polyA	0,00000
ddPCR_DNase_denat_CMV_enhancer	qPCR_DNase_polyA	1,00000
ddPCR_DNase_denat_polyA	ddPCR_DNase_denat_restr_beta_actin	0,00000
ddPCR_DNase_denat_polyA	ddPCR_DNase_denat_restr_beta_globin	0,00000
ddPCR_DNase_denat_polyA	ddPCR_DNase_denat_restr_CMV_enhancer	0,25810
ddPCR_DNase_denat_polyA	ddPCR_DNase_denat_restr_polyA	0,00000
ddPCR_DNase_denat_polyA	ddPCR_DNase_polyA	0,00000
ddPCR_DNase_denat_polyA	qPCR_DNase_beta_actin	0,00000
ddPCR_DNase_denat_polyA	qPCR_DNase_beta_globin	0,00000
ddPCR_DNase_denat_polyA	qPCR_DNase_CMV_enhancer	0,00000
ddPCR_DNase_denat_polyA	qPCR_DNase_denat_beta_actin	0,00000
ddPCR_DNase_denat_polyA	qPCR_DNase_denat_beta_globin	0,00000
ddPCR_DNase_denat_polyA	qPCR_DNase_denat_CMV_enhancer	0,99040
ddPCR_DNase_denat_polyA	qPCR_DNase_denat_polyA	0,00000
ddPCR_DNase_denat_polyA	qPCR_DNase_denat_restr_beta_actin	0,00000
ddPCR_DNase_denat_polyA	qPCR_DNase_denat_restr_beta_globin	0,00000
ddPCR_DNase_denat_polyA	qPCR_DNase_denat_restr_CMV_enhancer	0,00019
ddPCR_DNase_denat_polyA	qPCR_DNase_denat_restr_polyA	0,00000
ddPCR_DNase_denat_polyA	qPCR_DNase_polyA	0,00000
ddPCR_DNase_denat_restr_beta_actin	ddPCR_DNase_denat_restr_beta_globin	0,59932
ddPCR_DNase_denat_restr_beta_actin	ddPCR_DNase_denat_restr_CMV_enhancer	0,00000
ddPCR_DNase_denat_restr_beta_actin	ddPCR_DNase_denat_restr_polyA	0,00000
ddPCR_DNase_denat_restr_beta_actin	ddPCR_DNase_polyA	0,00000
ddPCR_DNase_denat_restr_beta_actin	qPCR_DNase_beta_actin	0,00000
ddPCR_DNase_denat_restr_beta_actin	qPCR_DNase_beta_globin	0,00000
ddPCR_DNase_denat_restr_beta_actin	qPCR_DNase_CMV_enhancer	0,00000
ddPCR_DNase_denat_restr_beta_actin	qPCR_DNase_denat_beta_actin	0,32484
ddPCR_DNase_denat_restr_beta_actin	qPCR_DNase_denat_beta_globin	0,20961
ddPCR_DNase_denat_restr_beta_actin	qPCR_DNase_denat_CMV_enhancer	0,00000
ddPCR_DNase_denat_restr_beta_actin	qPCR_DNase_denat_polyA	0,00002
ddPCR_DNase_denat_restr_beta_actin	qPCR_DNase_denat_restr_beta_actin	0,00000
ddPCR_DNase_denat_restr_beta_actin	qPCR_DNase_denat_restr_beta_globin	0,00000
ddPCR_DNase_denat_restr_beta_actin	qPCR_DNase_denat_restr_CMV_enhancer	0,00231
ddPCR_DNase_denat_restr_beta_actin	qPCR_DNase_denat_restr_polyA	0,00000
ddPCR_DNase_denat_restr_beta_actin	qPCR_DNase_polyA	0,00000
ddPCR_DNase_denat_restr_beta_globin	ddPCR_DNase_denat_restr_CMV_enhancer	0,00000
ddPCR_DNase_denat_restr_beta_globin	ddPCR_DNase_denat_restr_polyA	0,11022
ddPCR_DNase_denat_restr_beta_globin	ddPCR_DNase_polyA	0,00000
ddPCR_DNase_denat_restr_beta_globin	qPCR_DNase_beta_actin	0,00000
ddPCR_DNase_denat_restr_beta_globin	qPCR_DNase_beta_globin	0,00000
ddPCR_DNase_denat_restr_beta_globin	qPCR_DNase_CMV_enhancer	0,00000
ddPCR_DNase_denat_restr_beta_globin	qPCR_DNase_denat_beta_actin	0,00000
ddPCR_DNase_denat_restr_beta_globin	qPCR_DNase_denat_beta_globin	0,00000
ddPCR_DNase_denat_restr_beta_globin	qPCR_DNase_denat_CMV_enhancer	0,00000
ddPCR_DNase_denat_restr_beta_globin	qPCR_DNase_denat_polyA	0,64027
ddPCR_DNase_denat_restr_beta_globin	qPCR_DNase_denat_restr_beta_actin	0,00000
ddPCR_DNase_denat_restr_beta_globin	qPCR_DNase_denat_restr_beta_globin	0,00000
ddPCR_DNase_denat_restr_beta_globin	qPCR_DNase_denat_restr_CMV_enhancer	0,00000
ddPCR_DNase_denat_restr_beta_globin	qPCR_DNase_denat_restr_polyA	0,00000
ddPCR_DNase_denat_restr_beta_globin	qPCR_DNase_polyA	0,00000

ddPCR_DNase_denat_restr_CMV_enhancer	ddPCR_DNase_denat_restr_polyA	0,00000
ddPCR_DNase_denat_restr_CMV_enhancer	ddPCR_DNase_polyA	0,00000
ddPCR_DNase_denat_restr_CMV_enhancer	qPCR_DNase_beta_actin	0,00251
ddPCR_DNase_denat_restr_CMV_enhancer	qPCR_DNase_beta_globin	0,00000
ddPCR_DNase_denat_restr_CMV_enhancer	qPCR_DNase_CMV_enhancer	0,00000
ddPCR_DNase_denat_restr_CMV_enhancer	qPCR_DNase_denat_beta_actin	0,00000
ddPCR_DNase_denat_restr_CMV_enhancer	qPCR_DNase_denat_beta_globin	0,00000
ddPCR_DNase_denat_restr_CMV_enhancer	qPCR_DNase_denat_CMV_enhancer	0,97393
ddPCR_DNase_denat_restr_CMV_enhancer	qPCR_DNase_denat_polyA	0,00000
ddPCR_DNase_denat_restr_CMV_enhancer	qPCR_DNase_denat_restr_beta_actin	0,00000
ddPCR_DNase_denat_restr_CMV_enhancer	qPCR_DNase_denat_restr_beta_globin	0,00000
ddPCR_DNase_denat_restr_CMV_enhancer	qPCR_DNase_denat_restr_CMV_enhancer	0,00000
ddPCR_DNase_denat_restr_CMV_enhancer	qPCR_DNase_denat_restr_polyA	0,00000
ddPCR_DNase_denat_restr_CMV_enhancer	qPCR_DNase_polyA	0,00000
ddPCR_DNase_denat_restr_polyA	ddPCR_DNase_polyA	0,00000
ddPCR_DNase_denat_restr_polyA	qPCR_DNase_beta_actin	0,00000
ddPCR_DNase_denat_restr_polyA	qPCR_DNase_beta_globin	0,00000
ddPCR_DNase_denat_restr_polyA	qPCR_DNase_CMV_enhancer	0,00000
ddPCR_DNase_denat_restr_polyA	qPCR_DNase_denat_beta_actin	0,00000
ddPCR_DNase_denat_restr_polyA	qPCR_DNase_denat_beta_globin	0,00000
ddPCR_DNase_denat_restr_polyA	qPCR_DNase_denat_CMV_enhancer	0,00000
ddPCR_DNase_denat_restr_polyA	qPCR_DNase_denat_polyA	0,99873
ddPCR_DNase_denat_restr_polyA	qPCR_DNase_denat_restr_beta_actin	0,00872
ddPCR_DNase_denat_restr_polyA	qPCR_DNase_denat_restr_beta_globin	0,00491
ddPCR_DNase_denat_restr_polyA	qPCR_DNase_denat_restr_CMV_enhancer	0,00000
ddPCR_DNase_denat_restr_polyA	qPCR_DNase_denat_restr_polyA	0,00000
ddPCR_DNase_denat_restr_polyA	qPCR_DNase_polyA	0,00000
ddPCR_DNase_polyA	qPCR_DNase_beta_actin	0,11522
ddPCR_DNase_polyA	qPCR_DNase_beta_globin	1,00000
ddPCR_DNase_polyA	qPCR_DNase_CMV_enhancer	1,00000
ddPCR_DNase_polyA	qPCR_DNase_denat_beta_actin	0,00000
ddPCR_DNase_polyA	qPCR_DNase_denat_beta_globin	0,00000
ddPCR_DNase_polyA	qPCR_DNase_denat_CMV_enhancer	0,00000
ddPCR_DNase_polyA	qPCR_DNase_denat_polyA	0,00000
ddPCR_DNase_polyA	qPCR_DNase_denat_restr_beta_actin	0,00000
ddPCR_DNase_polyA	qPCR_DNase_denat_restr_beta_globin	0,00000
ddPCR_DNase_polyA	qPCR_DNase_denat_restr_CMV_enhancer	0,00000
ddPCR_DNase_polyA	qPCR_DNase_denat_restr_polyA	0,00000
ddPCR_DNase_polyA	qPCR_DNase_polyA	1,00000
qPCR_DNase_beta_actin	qPCR_DNase_beta_globin	0,77216
qPCR_DNase_beta_actin	qPCR_DNase_CMV_enhancer	0,77216
qPCR_DNase_beta_actin	qPCR_DNase_denat_beta_actin	0,00000
qPCR_DNase_beta_actin	qPCR_DNase_denat_beta_globin	0,00000
qPCR_DNase_beta_actin	qPCR_DNase_denat_CMV_enhancer	0,00000
qPCR_DNase_beta_actin	qPCR_DNase_denat_polyA	0,00000
qPCR_DNase_beta_actin	qPCR_DNase_denat_restr_beta_actin	0,00000
qPCR_DNase_beta_actin	qPCR_DNase_denat_restr_beta_globin	0,00000
qPCR_DNase_beta_actin	qPCR_DNase_denat_restr_CMV_enhancer	0,00000
qPCR_DNase_beta_actin	qPCR_DNase_denat_restr_polyA	0,00000
qPCR_DNase_beta_actin	qPCR_DNase_polyA	0,77216
qPCR_DNase_beta_globin	qPCR_DNase_CMV_enhancer	1,00000
qPCR_DNase_beta_globin	qPCR_DNase_denat_beta_actin	0,00000
qPCR_DNase_beta_globin	qPCR_DNase_denat_beta_globin	0,00000
qPCR_DNase_beta_globin	qPCR_DNase_denat_CMV_enhancer	0,00000
qPCR_DNase_beta_globin	qPCR_DNase_denat_polyA	0,00000
qPCR_DNase_beta_globin	qPCR_DNase_denat_restr_beta_actin	0,00000
qPCR_DNase_beta_globin	qPCR_DNase_denat_restr_beta_globin	0,00000
qPCR_DNase_beta_globin	qPCR_DNase_denat_restr_CMV_enhancer	0,00000
qPCR_DNase_beta_globin	qPCR_DNase_denat_restr_polyA	0,00000
qPCR_DNase_beta_globin	qPCR_DNase_polyA	1,00000
qPCR_DNase_CMV_enhancer	qPCR_DNase_denat_beta_actin	0,00000
qPCR_DNase_CMV_enhancer	qPCR_DNase_denat_beta_globin	0,00000
qPCR_DNase_CMV_enhancer	qPCR_DNase_denat_CMV_enhancer	0,00000
qPCR_DNase_CMV_enhancer	qPCR_DNase_denat_polyA	0,00000
qPCR_DNase_CMV_enhancer	qPCR_DNase_denat_restr_beta_actin	0,00000

qPCR_DNase_CMV_enhancer	qPCR_DNase_denat_restr_beta_globin	0,00000
qPCR_DNase_CMV_enhancer	qPCR_DNase_denat_restr_CMV_enhancer	0,00000
qPCR_DNase_CMV_enhancer	qPCR_DNase_denat_restr_polyA	0,00000
qPCR_DNase_CMV_enhancer	qPCR_DNase_polyA	1,00000
qPCR_DNase_denat_beta_actin	qPCR_DNase_denat_beta_globin	1,00000
qPCR_DNase_denat_beta_actin	qPCR_DNase_denat_CMV_enhancer	0,00000
qPCR_DNase_denat_beta_actin	qPCR_DNase_denat_polyA	0,00000
qPCR_DNase_denat_beta_actin	qPCR_DNase_denat_restr_beta_actin	0,00000
qPCR_DNase_denat_beta_actin	qPCR_DNase_denat_restr_beta_globin	0,00000
qPCR_DNase_denat_beta_actin	qPCR_DNase_denat_restr_CMV_enhancer	0,88860
qPCR_DNase_denat_beta_actin	qPCR_DNase_denat_restr_polyA	0,00000
qPCR_DNase_denat_beta_actin	qPCR_DNase_polyA	0,00000
qPCR_DNase_denat_beta_globin	qPCR_DNase_denat_CMV_enhancer	0,00000
qPCR_DNase_denat_beta_globin	qPCR_DNase_denat_polyA	0,00000
qPCR_DNase_denat_beta_globin	qPCR_DNase_denat_restr_beta_actin	0,00000
qPCR_DNase_denat_beta_globin	qPCR_DNase_denat_restr_beta_globin	0,00000
qPCR_DNase_denat_beta_globin	qPCR_DNase_denat_restr_CMV_enhancer	0,96181
qPCR_DNase_denat_beta_globin	qPCR_DNase_denat_restr_polyA	0,00000
qPCR_DNase_denat_beta_globin	qPCR_DNase_polyA	0,00000
qPCR_DNase_denat_CMV_enhancer	qPCR_DNase_denat_polyA	0,00000
qPCR_DNase_denat_CMV_enhancer	qPCR_DNase_denat_restr_beta_actin	0,00000
qPCR_DNase_denat_CMV_enhancer	qPCR_DNase_denat_restr_beta_globin	0,00000
qPCR_DNase_denat_CMV_enhancer	qPCR_DNase_denat_restr_CMV_enhancer	0,00000
qPCR_DNase_denat_CMV_enhancer	qPCR_DNase_denat_restr_polyA	0,00000
qPCR_DNase_denat_CMV_enhancer	qPCR_DNase_polyA	0,00000
qPCR_DNase_denat_polyA	qPCR_DNase_denat_restr_beta_actin	0,00000
qPCR_DNase_denat_polyA	qPCR_DNase_denat_restr_beta_globin	0,00000
qPCR_DNase_denat_polyA	qPCR_DNase_denat_restr_CMV_enhancer	0,00000
qPCR_DNase_denat_polyA	qPCR_DNase_denat_restr_polyA	0,00000
qPCR_DNase_denat_polyA	qPCR_DNase_polyA	0,00000
qPCR_DNase_denat_restr_beta_actin	qPCR_DNase_denat_restr_beta_globin	1,00000
qPCR_DNase_denat_restr_beta_actin	qPCR_DNase_denat_restr_CMV_enhancer	0,00000
qPCR_DNase_denat_restr_beta_actin	qPCR_DNase_denat_restr_polyA	0,00000
qPCR_DNase_denat_restr_beta_actin	qPCR_DNase_polyA	0,00000
qPCR_DNase_denat_restr_beta_globin	qPCR_DNase_denat_restr_CMV_enhancer	0,00000
qPCR_DNase_denat_restr_beta_globin	qPCR_DNase_denat_restr_polyA	0,00000
qPCR_DNase_denat_restr_beta_globin	qPCR_DNase_polyA	0,00000
qPCR_DNase_denat_restr_CMV_enhancer	qPCR_DNase_denat_restr_polyA	0,00000
qPCR_DNase_denat_restr_CMV_enhancer	qPCR_DNase_polyA	0,00000
qPCR_DNase_denat_restr_polyA	qPCR_DNase_polyA	0,00000
