# Supplementary material for: Accurate Quantification and Characterization of Adeno-Associated Viral Vectors
Source: Front Microbiol. 2019 Jul 17;10:1570. doi: 10.3389/fmicb.2019.01570 (PMC6650692; doi:10.3389/fmicb.2019.01570)
Supplement: Supplementary file 2 [file Data_Sheet_2.pdf]

Supplementary Material – Data Sheet 2

Accurate quantification and characterization of adeno-associated viral vectors

David Dobnik*, Polona Kogovšek, Tjaša Jakomin, Nejc Košir, Magda Tušek Žnidarič, Maja Leskovec, Stephen M. Kaminsky, Janet Mostrom, Hyunmi Lee, Maja Ravnikar

*** Correspondence:** David Dobnik: david.dobnik@nib.si

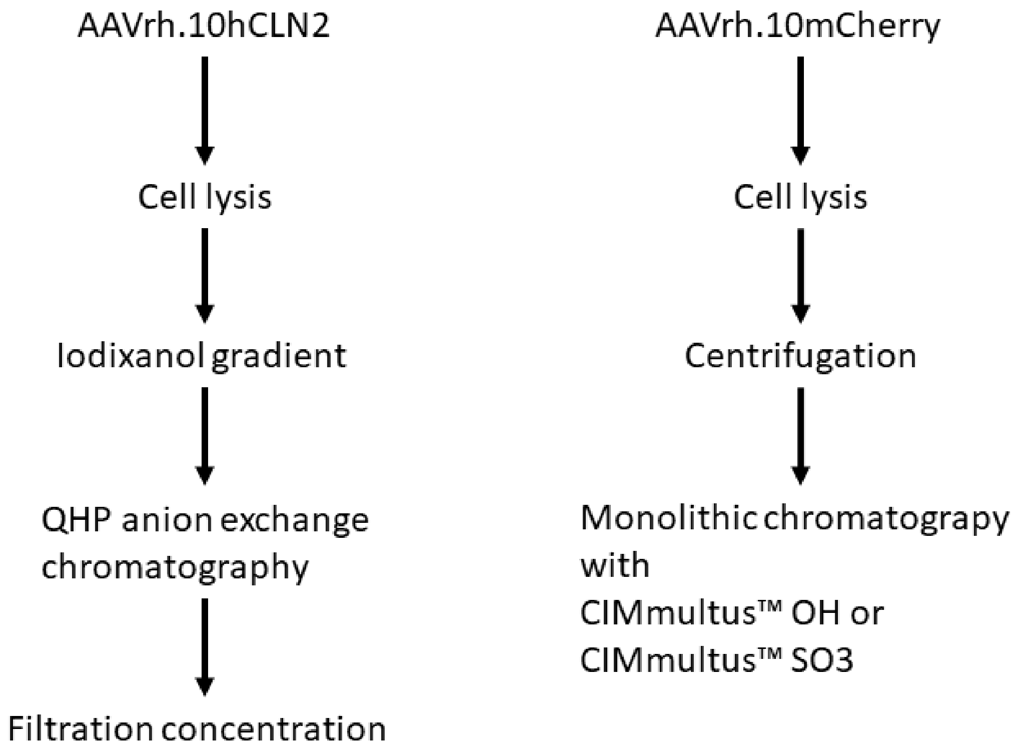

Supplementary Figure 1. Schematic representation of purification steps for AAVrh.10hCLN2 and AAVrh.10mCherry viral vectors

dG = -322.78 at 60 °C

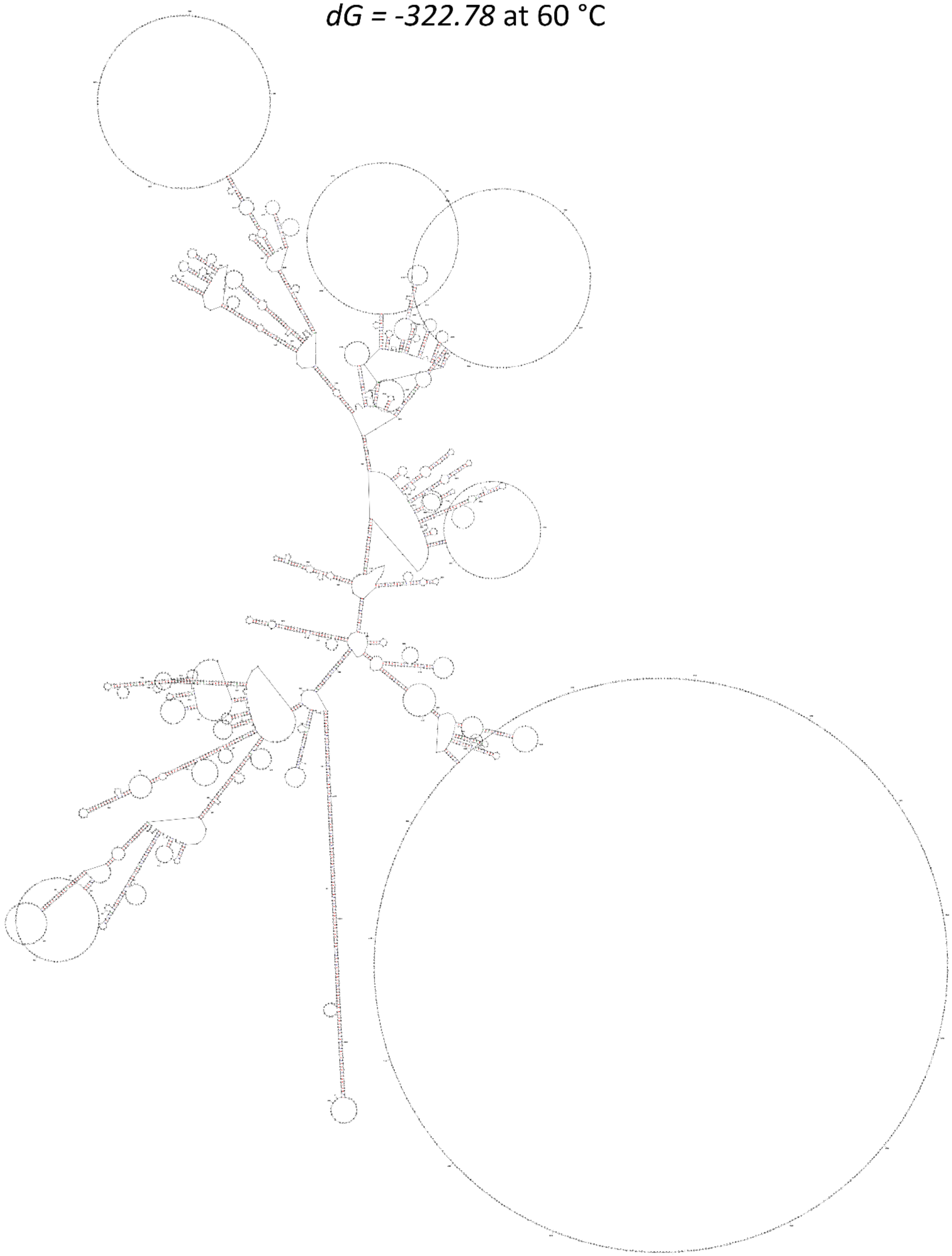

Supplementary Figure 2. Predicted secondary structure of vector construct at 60 °C.

dG = -230.93 at 60°C

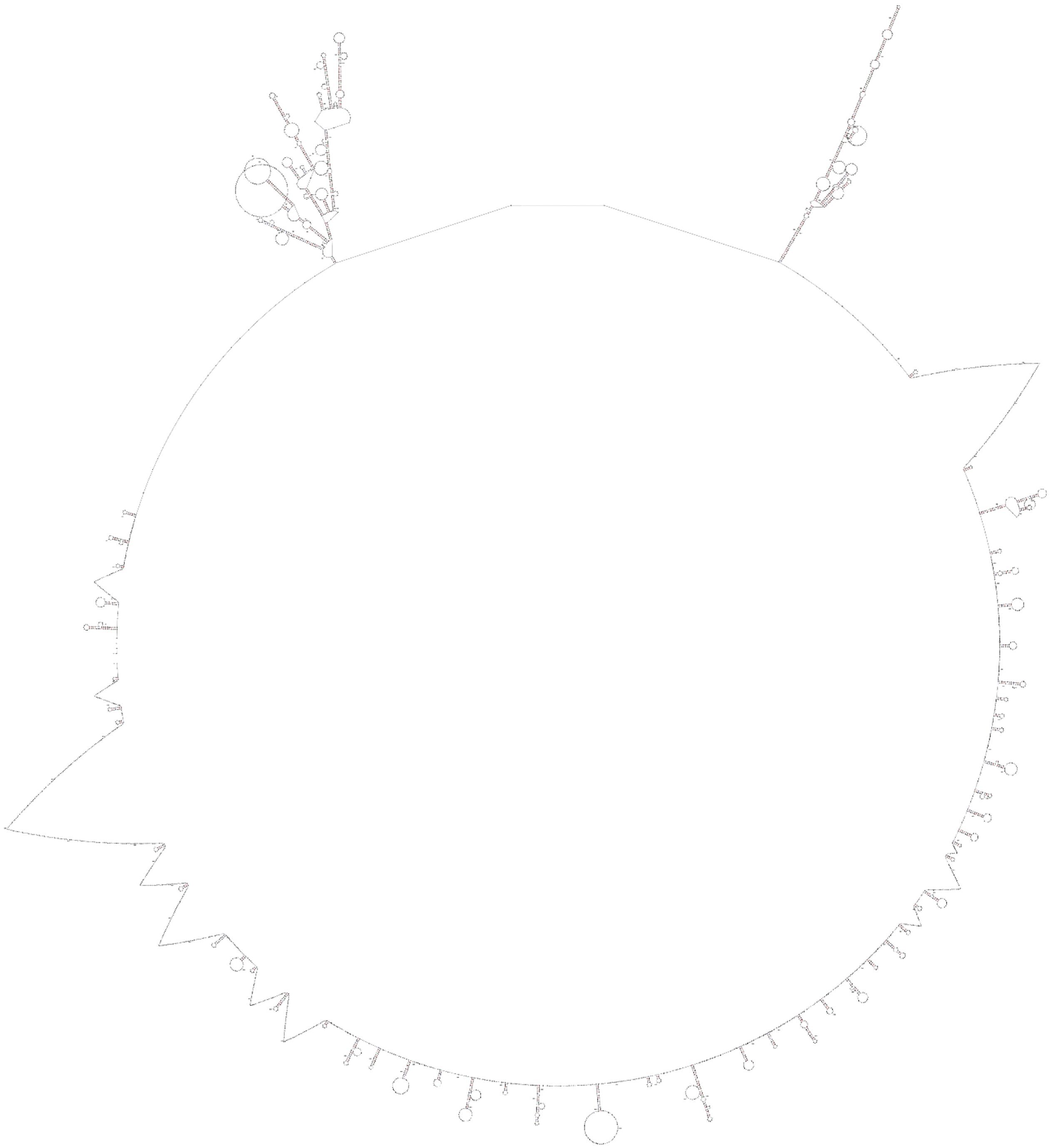

Supplementary Figure 3. Predicted secondary structure of vector construct without ITR regions at 60 °C.

dG = -15.76 at 99 °C

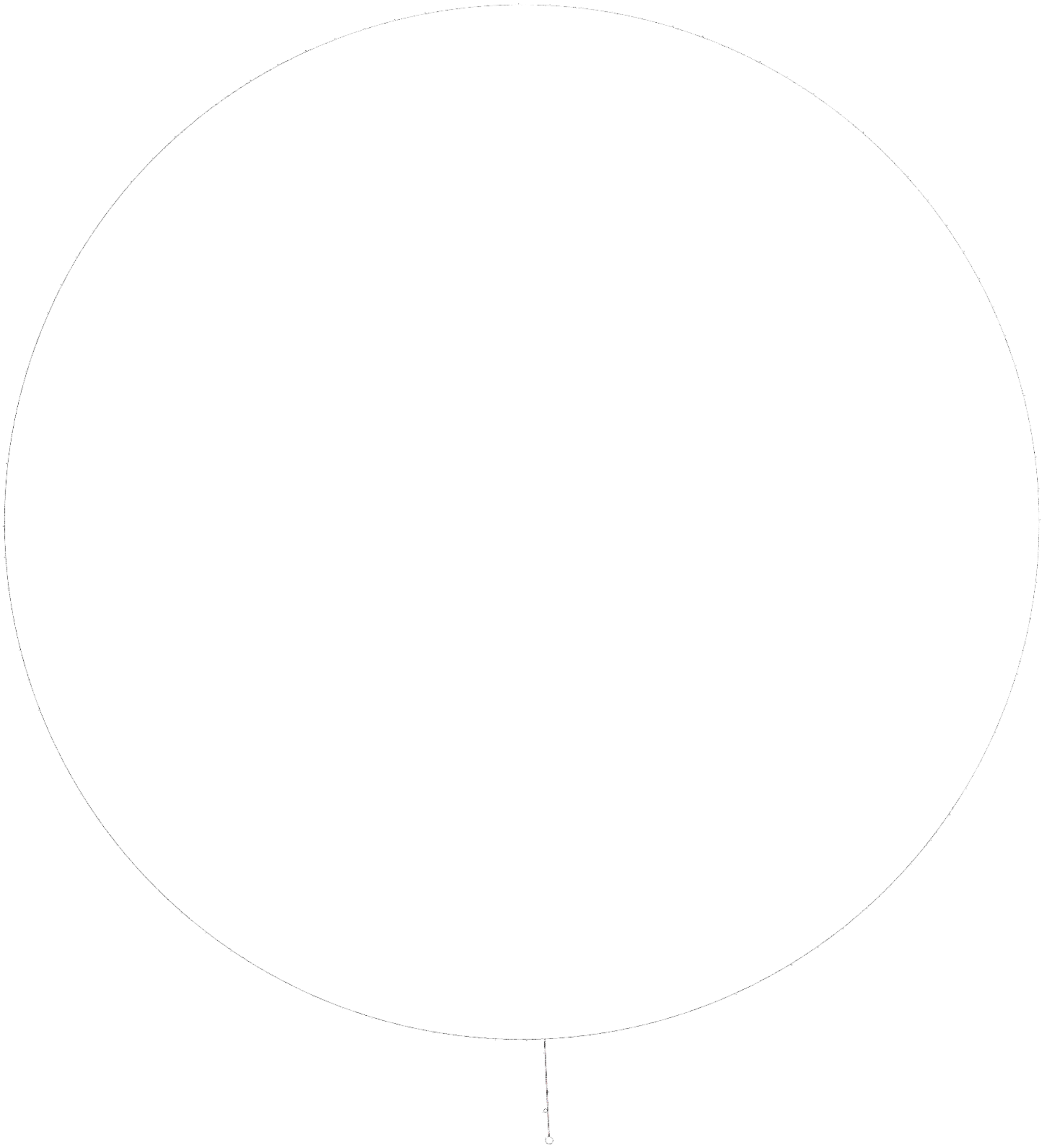

Supplementary Figure 4. Predicted secondary structure of vector construct at 99 °C.
